# Be Aware of Transient Dissolution Processes in Co_3_O_4_ Acidic Oxygen Evolution Reaction Electrocatalysts

**DOI:** 10.1021/jacs.4c14952

**Published:** 2025-01-15

**Authors:** Tatiana Priamushko, Evanie Franz, Anja Logar, Lazar Bijelić, Patrick Guggenberger, Daniel Escalera-López, Matej Zlatar, Jörg Libuda, Freddy Kleitz, Nejc Hodnik, Olaf Brummel, Serhiy Cherevko

**Affiliations:** † Helmholtz-Institute Erlangen-Nürnberg for Renewable Energy (IET-2), Forschungszentrum Jülich, 91058 Erlangen, Germany; ‡ Interface Research and Catalysis, ECRC, Friedrich-Alexander-Universität Erlangen-Nürnberg, 91058 Erlangen, Germany; § Department of Materials Chemistry, National Institute of Chemistry, 1000 Ljubljana, Slovenia; ∥ University of Nova Gorica, Vipavska 13, 5000 Nova Gorica, Slovenia; ⊥ Department of Functional Materials and Catalysis, Faculty of Chemistry, University of Vienna, 1090 Vienna, Austria; # Vienna Doctoral School in Chemistry (DoSChem), University of Vienna, 1090 Vienna, Austria

## Abstract

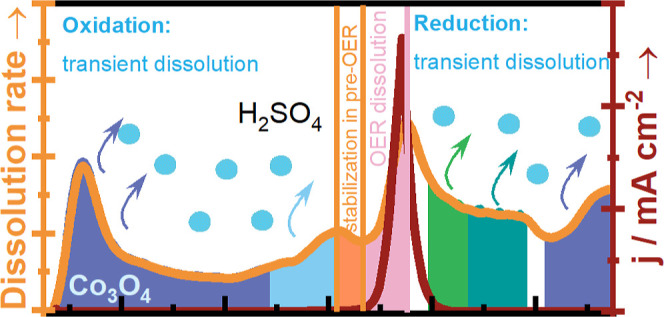

Recently, cobalt-based oxides have received considerable attention as an alternative to expensive and scarce iridium for catalyzing the oxygen evolution reaction (OER) under acidic conditions. Although the reported materials demonstrate promising durability, they are not entirely intact, calling for fundamental research efforts to understand the processes governing the degradation of such catalysts. To this end, this work studies the dissolution mechanism of a model Co_3_O_4_ porous catalyst under different electrochemical conditions using online inductively coupled plasma mass spectrometry (online ICP-MS), identical location scanning transmission electron microscopy (IL-STEM), and differential electrochemical mass spectrometry (DEMS). Despite the high thermodynamics tendency reflected in the Pourbaix diagram, it is shown that the cobalt dissolution kinetics is sluggish and can be lowered further by modifying the electrochemical protocol. For the latter, identified in this study, several (electro)chemical reaction pathways that lead to the dissolution of Co_3_O_4_ must be considered. Hence, this work uncovers the transient character of cobalt dissolution and provides valuable insights that can help to understand the promising stability of cobalt-based materials in already published works and facilitate the knowledge-driven design of novel, stable, abundant catalysts toward the OER in an acidic environment.

## Introduction

The intermittent nature of renewable energy sources requires the conversion of the produced energy into valuable chemicals, such as hydrogen, carbon monoxide, and small organic molecules.^1^ Water and CO_2_ electrolyzers offer efficient energy conversion and production of value-added chemicals and energy carriers.^2−4^ Although technologies like proton exchange membrane (PEM) and anion exchange membrane (AEM) electrolyzers are advancing rapidly, both suffer from design-dependent limitations. PEM electrolyzers, which are more promising in practical applications due to the high current densities and commercially available proton exchange membranes, are limited by highly aggressive acidic environments.^1,3^ While substantial advances have been made in the development of this technology, the critical bottleneck still lies in the oxygen evolution reaction (OER), which takes place at the anode side.^1,3^ Driving the OER in a PEM water electrolyzer requires the employment of expensive and scarce noble metals such as Ir,^5^ since cheaper and more abundant materials are considered unstable at low pH and high anodic potential. Nonetheless, recent studies suggested that Co- and Mn-based oxides can be relatively stable in acidic media and facilitate the OER reaction.^6−9^ Many strategies were reported to improve not only the activity but also the electrochemical stability of the non-noble metal-based catalysts. The most popular approaches include the development of protective layers on the anode catalysts,^10−12^ the utilization of core–shell structures,^13^ the control of the valence states of Co cations by altering the catalyst’s structure via doping or partially substituting its cations or anions with other elements,^14−26^ and the introduction of Supporting Information.^27−29^

Although considerable progress has been achieved in these studies, the degradation mechanisms of such catalysts are rarely investigated and/or understood. The stability of the catalysts is often analyzed only from the electrocatalytic performance point of view (e.g., stable performance during the chronopotentiometry or chronoamperometry measurements), while the structural and chemical stability are rarely considered. This can result in an overestimated evaluation of the catalysts’ durability, as the activity might be maintained simply by the constant dissolution of the catalyst, which would provide freshly exposed active sites during the operation until the complete degradation of the catalyst. Moreover, restructuring of the catalyst surface and initial dissolution of the electrodes, when first brought in contact with the highly oxidizing electrolyte, is usually not discussed. Therefore, the investigation of the dissolution and other possible degradation processes of the proposed potentially stable catalysts at low pH and under various electrochemical conditions is crucial.^9^ However, it is usually challenging and costly to study these processes as these experiments require the use of complex *in situ* techniques.^30^ Moreover, exploring the dissolution processes becomes significantly more complex with the number of elements in the catalyst going up.

We, therefore, suggest taking a step back and starting by analyzing the possible stability and degradation processes of simple systems such as single metal oxides, more specifically, model porous Co_3_O_4_. This will provide the necessary insights for a knowledge-driven design of the Co-based acidic OER catalysts. In this work, we investigate the behavior of ordered mesoporous (OM-) Co_3_O_4_ under various electrochemical conditions at pH ≈ 1. To do this, we employed online inductively coupled plasma mass spectrometry (online ICP-MS) using a scanning flow cell (SFC), identical location scanning transmission electron microscopy (IL-STEM), and differential electrochemical mass spectrometry (DEMS). This allows us to quantitatively analyze the dissolution of cobalt ions *in situ*,^31^ follow the morphological changes in the material during the dissolution and identify the degradation mechanism of the catalyst.

## Results and Discussion

Before discussing the dissolution data, we briefly describe the structure and morphology of the material utilized in this study (see Suppoting Information). Briefly, Co_3_O_4_ is a well-known spinel material where cobalt cations are present in two oxidation states: Co^II^ and Co^III^ (Figure S1).^32^ The material used here is OM-Co_3_O_4_ with a high specific surface area (108 m^2^ g^–1^, Figure S2a), narrow pore size distribution with most pores being 4.9 nm in size (Figure S2b), and an ordered mesoporous structure.^33−36^Figure S2c,d confirms the spinel structure of the material and the presence of typically observed oxidation states of Co (Co^II^ and Co^III^). In the past decade, it was also extensively studied as an efficient alkaline OER catalyst.^34,35^ All the experimental details, including synthesis, characterization, and electrochemical testing, are included in the Experimental Methods section.

**Figure 1:**
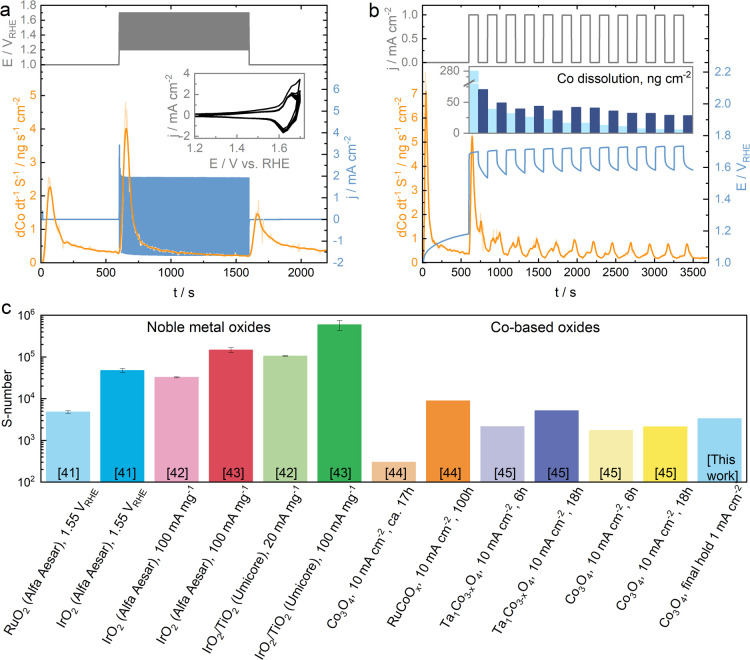
Dissolution profile of cobalt in Co_3_O_4_ under the AST (a) and start-up/shutdown (b) protocols. The inset in (a) shows the CVs recorded at 200 mV s^–1^ during the experiment. The inset in (b) presents the integrated dissolution peaks in the start-up/shutdown protocol (light/dark blue columns at inset). The light orange color represents the original data, and the dark orange shows the smoothed signal for a better presentation. All the current densities are obtained by normalizing the collected current by the geometric surface area of the electrode. Comparison of *S*-number between Co-, Ru-, and Ir-based materials (c). The *S*-number values for Ru and Ir-based materials were taken from the following works: Zlatar et al., 2023;^41^ Van Pham et al., 2020;^42^ Hoffmeister et al., 2024.^43^ The *S*-number values for Co-based materials were taken from the following works: Zhu et al., 2023^44^ and Lee et al., 2024.^45^ The numbers in the columns refer to the number of references cited.

The investigation of Co_3_O_4_ stability in acidic electrolytes under varied electrochemical conditions started with specifically designed protocols based on the initial dissolution assessments (see Figure S3). Accelerated stress tests (ASTs) with different upper and lower potential limits (UPL and LPL, respectively) are presented in Figures 1a and S4a–c. The first dissolution peak is observed at the very first contact of the electrode and the electrolyte, which is common for many materials.^37−39^ With the start of a rapid scanning (200 mV s^–1^) in the potential window of the oxidation and reduction reactions (possibly Co^II/III^ and Co^III/III–IV^ redox couples, 1.2–1.65 *V*_RHE_) and even a start of the OER (up to 1.7 *V*_RHE_), we detected a significant dissolution of the cobalt oxide, as can be seen in Figure 1a. Interestingly, the dissolution decreases, and the Co dissolution rate drops to the level of a steadily decreasing signal from the contact peak within ca. 50 cycles (marked as a dashed blue line in Figure S4a–c) suggesting the material’s passivation or stabilization. Such a drastic dissolution at the start of cycling can be caused not by cycling itself but by the sharp initial increase in the applied potential, which causes prompt oxidation of the surface and, thus, transient dissolution due to the surface restructuring.^40^ The dissolution profile depicted in Figure S4d supports this assumption, as the cobalt signal significantly rises with every large swap in the applied potential and decreases quickly when the potential is held constant. We assume that at a constant potential, the Co_3_O_4_/electrolyte interface reaches an equilibrium-like state relatively fast, presumably due to the passivation of the surface, while any significant change in the potential triggers the rapid dissolution of cobalt. This trend seems to be an intrinsic property of Co_3_O_4_, as such behavior is seen for each AST protocol regardless of the potential at which the sample was exposed to the electrolyte or the potential window in which the cycling was performed.

Steep potential changes usually occur during the start-up/shutdown (power on/off) operation of the PEMWE cells and prolonged cell-off times, which, based on the previously discussed findings, would cause a dramatic dissolution of the Co_3_O_4_ catalyst at the anode. To examine how damaging the fluctuating energy input can be for cobalt oxide, we designed an electrochemical protocol shown in Figure 1b. The catalyst was first contacted at the open circuit potential (OCP) (0 mA cm^–2^) and held for 10 min to allow the cobalt signal to decrease for a better resolution of the following dissolution peaks. After that, the material was exposed to 12 consecutive start–stop cycles from 1 mA cm^–2^ to OCP with the steps of 2 min in time. Predictably, the highest dissolution peak (after the contact peak) is observed at the start of the first cycle, when the potential increases from ca. 1.2 *V*_RHE_ to ca. 1.75 *V*_RHE_. Even within the 2 min hold, however, cobalt dissolution decreases drastically due to the stabilization of the interface under constant conditions (Figure S5a). A second dissolution peak is observed at the power-off point, where the potential changes cathodically. The inset in Figure 1b demonstrates the dissolution of cobalt obtained from dissolution rate peak integration. The dissolution of cobalt triggered by the anodic change in the potential, although extremely high at the start of the protocol, decreases continuously with each subsequent cycle and is lower than cathodic dissolution (dissolution during the OCP, termed this way for the sake of simplicity, even though the exact dissolution mechanism may be anodic, see below) already during the second cycle. One can assume that cobalt species with higher oxidation states, possibly CoO_2_, formed during the oxidation of Co^III^- to Co^III–IV^-based species in the precatalytic region (the potential window in this protocol) are stable. It is important to note that it is impossible at this point to draw conclusions about the oxidation states of the formed cobalt species, as studies with the use of spectroscopic techniques are needed to answer this question. It seems that these formed species with higher oxidation states can be stable even in the OER region as long as the potential is held constant, and the current density is low. The amount of cathodically dissolved cobalt, however, hovers around 30–40 ng cm^–2^ per hold after the second cycle until the end of the protocol. The previously formed Co^III–IV^-based species seem to dissolve (partially) rapidly during each cathodic sweep, presumably due to their reduction. Such behavior suggests that the Co^III^ to Co^III–IV^ oxidation process is less destructive than the respective reduction reaction, and either not all the oxidized species are reduced during each following cathodic sweep or not all reduction acts cause dissolution (Figure S5b). Following this, the stability number (*S*-number)^46^ of the cobalt oxide increases with each cycle of the protocol (Figure S5c). We also compared the *S*-number obtained in this work with the other reported materials (Figure 1c). Naturally, the Co-based catalysts exhibit lower stability performance, but some mixed metal oxides are already approaching the *S*-number of pure ruthenium oxide. Moreover, even the stability of Ir-based OER catalysts depends on their crystal and morphological structure, greatly affecting the *S*-number that can vary from 300 to 320,500, as was shown by Maillard et al.^47^

**Figure 2:**
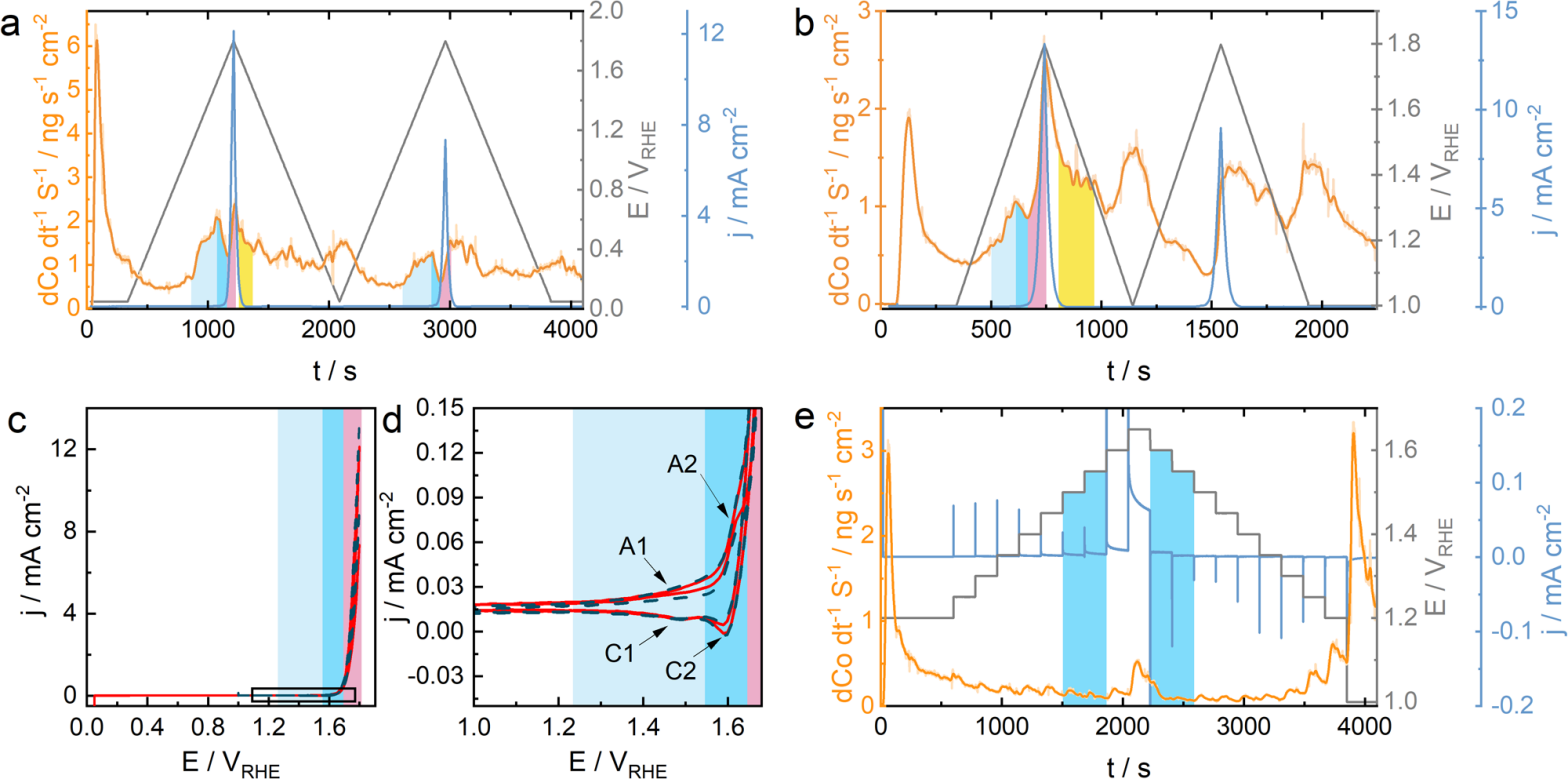
Dissolution profiles of cobalt in Co_3_O_4_ during the CV measurements with the LPL of 0.05 *V*_RHE_ (a) and 1.00 *V*_RHE_ (b) at 2 mV s^–1^ scan rate; comparison of the voltammograms recorded during these experiments on a full scale (c), and the zoomed-in area of the redox peaks (d); the dissolution profile of Co during the stepwise potentiostatic test (e). All the current densities are obtained by normalizing the collected current by the geometric surface area of the electrode. The light blue color in the CV plots highlights the region of the first transient dissolution due to the oxidation reactions, blue highlights the stabilization precatalytic region, pink highlights the OER-induced dissolution, and yellow highlights the transient dissolution due to the reduction reactions. The lowest dissolution regions in (e) are also highlighted in blue.

To provide more information about the relationship between the redox processes and the dissolution of cobalt, it is crucial to examine the behavior of Co_3_O_4_ during the continuously changing potential. Figure 2a,b presents the dissolution profiles of cobalt recorded during the CV measurements starting at either 0.05 *V*_RHE_ or 1.00 *V*_RHE_ LPL. To successfully resolve the dissolution peaks triggered by numerous possible redox reactions, we performed these CV measurements at a low scan rate of 2 mV s^–1^. The respective voltammograms are presented in Figure 2c,d. Although we observe several dissolution peaks, we will focus mostly on a few of them, which we highlight in colors on the graphs. To explain the dissolution trends, we also suggest possible reaction pathways for the processes discussed here, which are presented in Chapter 3 of the Supporting Information and discussed in detail later in the text. The relatively rapidly increasing dissolution of cobalt highlighted in light blue appears in a wide potential window, where, following the CV redox peaks, the first oxidation reaction (A1, possibly oxidation of Co^II^ to Co^III^) takes place (eq A1 in the Supporting Information). The next oxidation reaction, presumably the transition to higher oxidation states (A2, Co^III^ to Co^III–IV^), occurs in the narrow potential window (ca. 1.55–1.65 *V*_RHE_) highlighted in blue (eq A2 in the Supporting Information). Interestingly, this reaction induces stabilization of cobalt oxide, as we observe a clear and prompt drop in the dissolution rate of cobalt. Hypothetically, the formation of stable CoO_2_ species might take place in the pre-OER region, which is suggested by the Pourbaix diagram of cobalt (Figure S6).^48^ However, the potential of the CoO_2_ formation can be shifted to this potential window at higher aqueous ion concentrations according to Figure S6, where we compare the change in the Pourbaix diagrams at the ion concentrations of 10^–3^, 10^–6^, and 10^–9^ M. Other feasibly formed species could be CoO(OH)_*x*_, which are typically formed on Co_3_O_4_ surface in alkaline media^49^ under similar conditions and which formation was recently reported by Natarajan et al. in acidic media.^32^ However, the formation of these species at low pH contradicts the thermodynamic data presented in the Pourbaix diagrams (Figure S6). This stabilization region is followed by the OER region (highlighted in pink, Figure 2), where the rapid dissolution starts. We assume that this dissolution is OER-induced, and a possible mechanism includes the decomposition of Co^III–IV^-based species to soluble low oxidation state species (e.g., CoO) with simultaneous liberation of O_2_, suggesting lattice participation (lattice OER, LOER; eqs B1–B1.2 in the Supporting Information).^50,51^ Note that online dissolution studies using ICP-MS can only indirectly suggest the mechanism of this reaction. Direct evidence using DEMS^31,52^ is discussed in detail at a later point. Returning to Figure 2, in the cathodic direction, the Co dissolution decreases rapidly, as also the rate of the OER decays. A further change of the potential in the cathodic direction (approximately 1.6–1.4 *V*_RHE_) triggers a slight increase in the dissolution rate of cobalt (highlighted in yellow in Figure 2a,b) due to the reduction of the previously oxidized Co species (Figure 2d; eqs C1 and C2 in the Supporting Information).

As we found that the precatalytic region stabilizes the Co_3_O_4_ in the acidic environment, we studied this region in more detail. To do this, we designed protocols where the potential changes in a stepwise manner. Figures 2e and S7 demonstrate the dissolution profiles of cobalt recorded during these electrochemical protocols. Here, we highlighted in blue the potential regions where the lowest dissolution is observed. Generally, when the potential increases with the step of 50 mV from 1.2 to 1.6 *V*_RHE_, a low dissolution rate is detected. Only when the potential exceeds 1.65 *V*_RHE_ does the observed dissolution peak increase significantly. When the potential changes cathodically, an even higher dissolution is triggered at potentials below 1.25 *V*_RHE_, which matches what we observed during the CV measurements (Figure 2a,b). As Figures S4d and S7 show, we experimented with the length and size of the steps in these protocols, which appeared to have a significant effect on the cobalt dissolution rate. The stability window of Co_3_O_4_ found in the stepwise protocols is slightly shifted in comparison to the one observed in the CV measurements (ca. 1.4–1.6 *V*_RHE_). However, cobalt dissolution can be minimized depending on the electrochemical measurements’ conditions, e.g., smaller potential steps or the difference between the initial and final potentials (Figure S7d). Nevertheless, the potential, which is high enough to start the OER and increase the current, leads to the dissolution of cobalt even at very low current densities.

**Figure 3:**
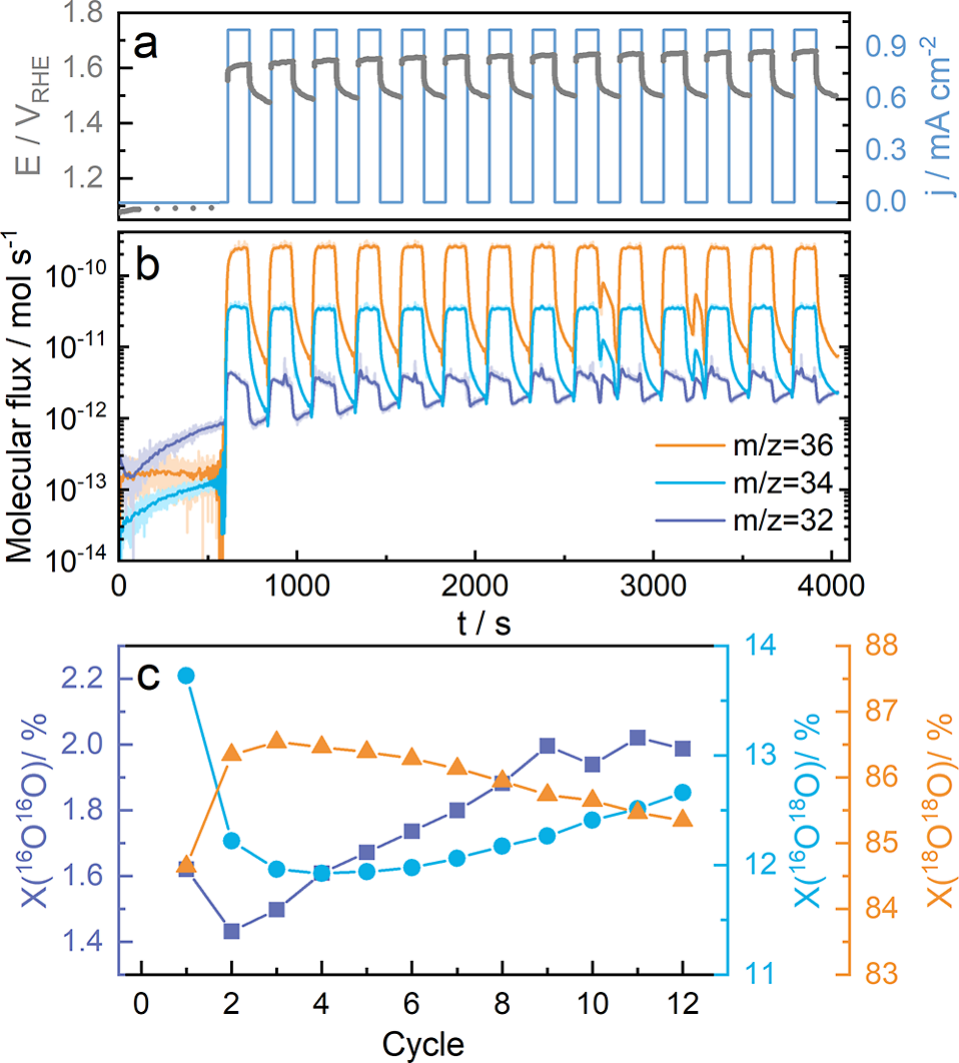
DEMS results for the start-up/shutdown protocol recorded with OM-Co_3_O_4_ in the 97% H_2_^18^O isotope-labeled 0.05 M H_2_SO_4_. The upper plot (a) depicts the applied current and recorded potential response; plot (b) shows the molecular flux of the ^18^O^18^O (*m*/*z* = 36), ^16^O^18^O (*m*/*z* = 34), and ^16^O^16^O (*m*/*z* = 32); plot (c) demonstrates the fractions of ^18^O^18^O (*m*/*z* = 36), ^16^O^18^O (*m*/*z* = 34), and ^16^O^16^O (*m*/*z* = 32) calculated for each cycle.

As previously mentioned, the OER-induced dissolution might occur due to the participation of the oxide layer in the OER when the exchange of oxygen atoms between the electrode and the electrolyte occurs. To estimate the role and extent of the oxygen exchange in Co_3_O_4_ during the acidic OER, we performed the start-up/shutdown protocol in the isotope-labeled electrolyte using DEMS. In Figure 3, we illustrate the performed protocol and the obtained DEMS results. Both *m*/*z* = 34 (^16^O^18^O) and *m*/*z* = 32 (^16^O^16^O) signals steadily increase during the initial OCP hold, implying the release of oxygen from the lattice of cobalt oxide due to its dissolution. In all consecutive cycles, we observe an increased O_2_ evolution under operation conditions (1 mA cm^–2^), while after the shutdown (0 mA cm^–2^), it decreases rapidly. However, the ratio between *m*/*z* = 36, *m*/*z* = 34, and *m*/*z* = 32 changes during the consecutive cycles. In the initial cycle, the *m*/*z* = 34 (^16^O^18^O) and *m*/*z* = 32 (^16^O^16^O) signals are higher than expected from the electrolyte content (97% H_2_^18^O) and decrease within a few first cycles (Figure S8). During further cycles, however, the trend changes, and the ^16^O^18^O and ^16^O^16^O fractions start rising again. We assume that the dissolution of Co_3_O_4_ during the first OCP step (also observed in Figure 1b) results in water (H_2_^16^O) formation (eqs OCP1 and OCP2, Supporting Information), which increases the ratio of H_2_^16^O to H_2_^18^O water near the electrode and originates the initial high fractions of *m*/*z* = 34 (^16^O^18^O) and *m*/*z* = 32 (^16^O^16^O) (note that, unlike flow conditions in the SFC, the electrolyte was stagnant in the DEMS cell). The depletion of the formed H_2_^16^O by consumption and diffusion decreases these fractions in the next few cycles. An increase of both fractions after cycle 4 suggests that oxygen exchange and lattice participation of cobalt oxide take place during the OER (Figure S8b, eqs B1, B1.1, and B1.2).

**Figure 4:**
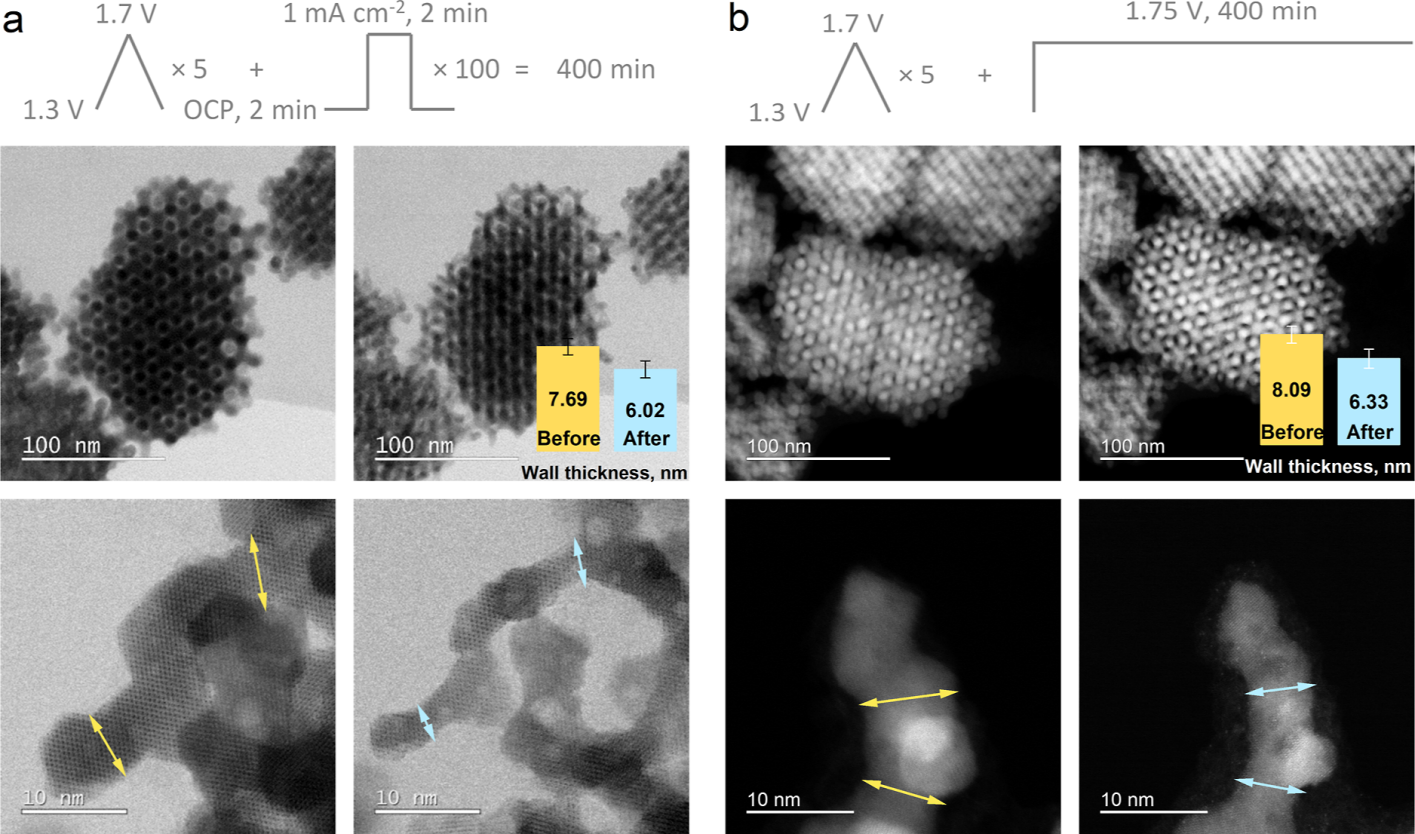
High-resolution annular bright field (ABF) and high angle annular dark field (HAADF) IL-STEM images of Co_3_O_4_ before and after the start-up/shutdown (a) and constant potentiostatic hold (b) electrochemical protocols.

Utilizing model porous Co_3_O_4_ with a well-defined ordered mesoporous structure has the benefit of more accurately tracking the morphological changes in the material under electrochemical conditions. Here, we employed IL-STEM to examine the dissolution of ordered mesoporous cobalt oxide visually. The images presented in Figure 4 confirm the dissolution of Co_3_O_4_ as the thickness of the catalyst walls decreases similarly after various electrochemical protocols. It is worth mentioning that the walls of such a complex 3D mesoporous structure seem to decrease in size uniformly, which suggests that most of the catalyst surface is in contact with the electrolyte, and this morphology does not prevent the electrolyte flow through the pores. Although the decrease in size of the catalyst confirms the dissolution of cobalt, it is obvious that the crystallinity of the oxide did not undergo significant changes, which contradicts some of the published works, where Co_3_O_4_ spinel (surface) structure was destroyed due to the exposure to the OER conditions.^53,54^ Interestingly, there is also no sign of a thin amorphous layer on the surface, which suggests impressive structural stability. Moreover, the lack of the amorphous layer seems to be independent of the electrochemical protocol. Amorphization of the cobalt oxide’s surface during the OER was first observed in alkaline media and is assumed to be beneficial as it occurs due to the formation of active CoO(OH) species.^35,55,56^ Yang’s group observed the formation of an amorphous layer on the Co_3_O_4_ surface also during the acidic OER, which they explained by the formation of the hydrous oxide layer (HOL).^32^ The authors concluded that the HOL growth damaged the crystalline integrity of Co_3_O_4_ and correlated its formation to the initial structure—higher amounts of Co^III^ in the Co_3_O_4_ structure are associated with the covalent bonding, which induces the LOER and creates a higher number of oxygen vacancies on the oxide’s surface promoting dissolution of cobalt. Although we certainly observe cobalt dissolution and assume the LOER mechanism, we do not see the formation of a thin amorphous layer in the high-resolution transmission electron microscopy (HR-TEM) images. The authors also suggested that the HOL formation speed and, thus, the dissolution of Co ions can be delayed by the presence of a higher number of Co^II^ species on the oxide’s surface.^32,45^ Higher Co^III^/Co^II^ ratio, however, is considered beneficial for the activity of Co_3_O_4_ in the acidic OER,^57,58^ which implies that the main challenge of using cobalt-based oxides as the acidic OER catalysts would be to find a middle ground between their activity and stability. This challenge, however, can also be addressed by facet engineering, as was shown previously.^59^ All this opens many directions for further research in this area.

Getting back to our results, Figure 4 suggests that overall Co_3_O_4_ dissolution is similar in both cases: at the constant potential and start-up/shutdown operation. This might be an indirect confirmation of the OER-induced dissolution, which again suggests lattice participation in the OER process. However, resolving the exact dissolution mechanisms during the OER would require other approaches, some of which were successfully employed in recently published papers.^7,30,45^ On the other hand, contact dissolution could play an important role in morphological changes. As we demonstrated above, bringing Co_3_O_4_ in contact with the electrolyte often contributes the most to its overall dissolution (Figures 1 and 2). Therefore, we also tracked the change in the OM-Co_3_O_4_ morphological structure right after its contact with the electrolyte without any applied electrochemical protocol (OCP). Figure S9 reveals an obvious change in the size of the catalyst due to its intense contact dissolution. A comparison of the images after the initial contact and after the two electrochemical protocols shown in Figure 4 suggests that almost 7 h long electrochemical protocols under constant or changing potentials in the OER region trigger lower dissolution than the initial material-electrolyte contact. This confirms that the potential-triggered transient dissolution due to the rapid oxidation processes on the Co_3_O_4_ surface is the main contributor to the degradation of the cobalt oxide in acidic electrolytes. It also offers several options for limiting the overall dissolution of Co_3_O_4_ during the operation. As an example, using a low volume of electrolyte may help to reach the saturation limit of Co ions in the solution and prevent further intense dissolution of the material.^9,60^ Moreover, even in the scope of this work, we have seen different intensities of the contact dissolution peaks depending on the conditions at which the contact occurred (e.g., OCP, 0.05, or 1 *V*_RHE_), which suggests that an optimal contacting condition might help to decrease the dissolution of the cobalt oxide catalyst. Another option to minimize contact dissolution would be to introduce species of high oxidation states on the surface of the oxide-based electrode to prevent additional oxidation reactions. This, however, needs further intensive investigation. On the other hand, as we mentioned earlier, a few works hypothesize that Co in the lower valence states is more stable and suggest doping the Co_3_O_4_ spinel with elements like Mo or W, which can serve as sources of electrons due to their multivalent nature and, therefore, prevent further oxidation of cobalt cations.^25,26,61,62^ This strategy was studied more actively for the Ni, Fe, and Co-based catalysts for the alkaline OER,^61^ and is only now being implemented for the acidic OER.^26,62^

**Figure 5:**
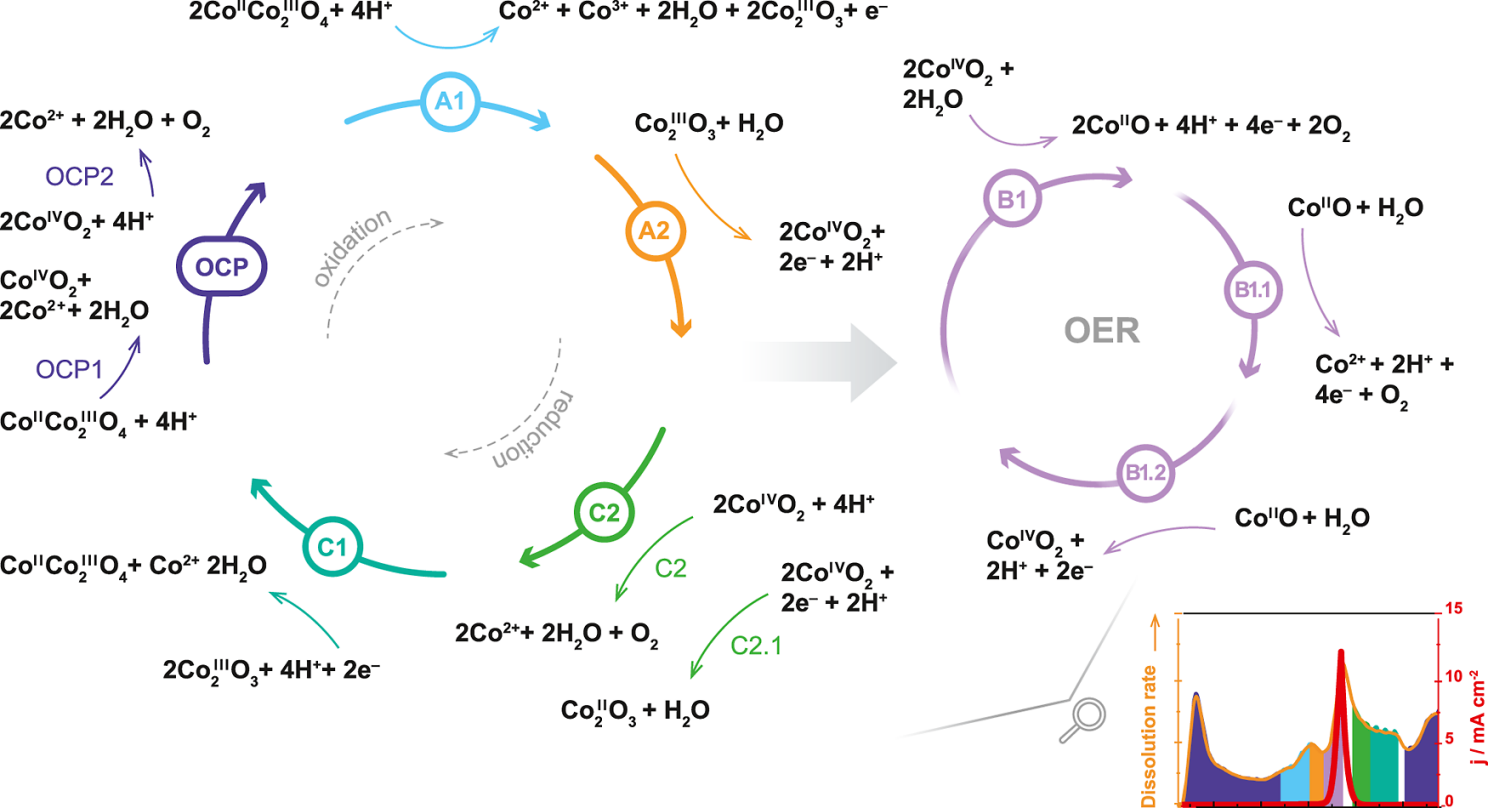
Schematic representation of the suggested mechanisms of the reactions occurring during the cyclic voltammetry at the Co_3_O_4_ surface. The color code of the arrows is respective to the highlighted regions in the dissolution profile presented in the bottom right corner of the scheme. Each reaction arrow is numbered according to the equations presented in Chapter 3 of the Supporting Information. Alternative reaction paths are not shown here for the simplicity of the figure. Copyright 2024 Kateryna Streltsova.

Based on this work’s results, we attempted to identify all the dissolution processes that occur, starting with the initial contact between the catalyst and the electrolyte and during the cycling from 1.0 to 1.8 *V*_RHE_. Figure 5 depicts the possible reactions schematically, and Chapter 3 in the Supporting Information offers the equations we propose to describe the observed behavior of cobalt oxide. We emphasize that our suggestions are based only on the results presented in this work and on the thermodynamic data. Future spectroscopic studies are necessary to improve our hypothesis. At first, an intense chemical dissolution occurs at the initial contact of the electrode with the electrolyte (violet region, eqs OCP1 and OCP2). This dissolution process is accompanied by the formation of water and oxygen, as the intense dissolution of cobalt ions frees a significant amount of oxygen ions from the spinel lattice, some of which then form water due to the interaction with H^+^ present in the electrolyte. Changing the potential into the anodic direction causes surface restructuring and the first oxidation reaction (A1 oxidation peak in Figure 2d, assumingly Co^II^ to Co^III^) in the range of approximately 1.3–1.55 *V*_RHE_. This oxidation reaction also results in cobalt dissolution (blue region, eq A1). Strictly speaking, the dissolution starts even before the oxidation region (before 1.4 *V*_RHE_). However, it is difficult to separate those dissolution peaks, and here, we highlight them as one. With further increase in potential, the second oxidation reaction starts (A2 in Figure 2d, Co^III^ to presumably Co^III–IV^), and the formation of stable Co^III–IV^-based species occurs (orange region, eq A2). This reaction does not trigger dissolution, as we can see a clear drop in the dissolution rate. We assume that these formed Co^III–IV^-based species can be stable at the constant potential or in a narrow potential window (1.55–1.65 *V*_RHE_). In our case, however, the potential increases further and enters the OER region, where the dissolution rate is the highest (pink region, eqs B1, B1.1, and B1.2). By analyzing the online ICP-MS and DEMS data, we assume that Co_3_O_4_ lattice participation in the OER might be the main reason for such a high dissolution of cobalt. During the OER, previously formed Co^III–IV^-based species decompose to unstable low oxidation states species of cobalt (Co^II^ species, e.g., CoO) with a simultaneous formation of O_2_ (eq B1), which is followed by the dissolution of cobalt and, again, the formation of O_2_ (eq B1.1). When the potential stays constant or decreases, the dissolution of cobalt drops, which, as we assume, happens due to the reoxidation of Co^II^-based species and stabilization of the surface (eq B1.2). We also suggest an alternative path for the LOER mechanism, which can be adapted for the Co^II^/Co^III^ redox couple if the formation of the Co^IV^ species is proven impossible by further studies and is presented by eqs B2.1–B2.4 in the Supporting Information. After reaching 1.8 *V*_RHE_, we switched to the cathodic direction and saw an immediate decrease in the dissolution of cobalt as there was no oxidation anymore. There are two dissolution areas presumably caused by the reduction processes (assumingly Co^III–IV^/Co^III^ (C2 reduction peak in Figure 2d) and Co^III^/Co^II^ redox couples (C1 in Figure 2d), green regions, eqs C1 and 2, respectively). Although it is difficult to confirm the formation of Co^IV^ species in the precatalytic region,^56^ the presence of the later redox peaks suggests the oxidation of Co^III^ to a higher oxidation state and is suggested by the thermodynamic data presented in Pourbaix diagrams (Figure S6).^32,44^

## Conclusion

In conclusion, our work provides valuable insights into the stability of highly porous spinel Co_3_O_4_. These results underline the promising performance of cobalt oxide in acidic electrolytes and highlight the critical importance of the conditions at which the Co-based electrode is brought in contact with the electrolyte and is further tested. We also found a potential stability window, which lies in the precatalytic region where the formation of stable Co^III–IV^-based species occurs (in our case, 1.55–1.65 *V*_RHE_ based on the 2 mV s^–1^ CVs). Analysis of the OER mechanism by the isotope-labeled experiments with DEMS and the IL-TEM results suggests lattice participation in the OER, which induces rapid dissolution of cobalt during the reaction. However, the overall OER-induced dissolution seems to be lower than the initial contact dissolution of cobalt oxide and plays a less destructive role in the long run. Moreover, based on the HR-TEM images, we conclude that the crystallinity of the material is not affected by the harsh oxidizing conditions. Finally, by correlating and analyzing all the results, we come up with the possible reaction mechanism. We believe that this work offers valuable insights that provide the fundament for further knowledge-driven research on non-noble metal-based oxides and on the optimal conditions for their testing as acidic OER catalysts. Much extensive research focused on developing alternative noble metal-free catalysts for PEM water electrolysis has been published recently, where the activity is the focus of the investigation. With this work, we hope to bring the community’s attention to the holistic approach to evaluating the electrocatalytic performance of the materials, where the dissolution plays a significant role in the stability and activity of the materials.

## Experimental Methods

### Synthesis of KIT-6-100 Ordered Mesoporous Silica

Mesoporous KIT-6 silica with a 3-D cubic *la*3*d* symmetry was prepared following the procedure reported by Kleitz et al.^63^ Briefly, 5.13 g of Pluronic P123 triblock copolymer (Sigma-Aldrich, Germany), 185.33 g of deionized water, and 9.92 g of concentrated HCl (37%, Sigma-Aldrich, Germany) were weighed into a 250 mL PP reaction flask. The P123 pore-generating agent was fully dissolved by vigorous stirring overnight at 35 °C in an incubator. The next day, 5.13 g of *n*-butanol (99%, Thermo Fisher Scientific, Germany) was added to the stirred mixture. After 1 h of stirring, 11.03 g of tetraethyl orthosilicate (TEOS, 98%, Thermo Fischer Scientific, Germany), the silica precursor, were added in one shot. After 24 h of vigorous stirring at 35 °C, the reaction flask was placed in a convection oven for hydrothermal treatment at 100 °C for 48 h in static conditions. The precipitated silica was isolated by filtration and dried overnight at 140 °C. Subsequently, the powder was dispersed in a mixture of 200 mL of ethanol and 2 drops of concentrated HCl for 45 min to remove the soft template. After filtration, the powder was calcined at 550 °C for 5 h.

### Preparation of Ordered Mesoporous Co_3_O_4_ via One-Step Impregnation Nanocasting Procedure

Based on the procedure described by Yen et al.,^64^ the KIT-6-100 hard template was dried at 150 °C in a vacuum oven overnight prior to use. 1.0 g of the silica was mixed with 2.5 g Co(NO_3_)_2_·6H_2_O (98.0–102.0%, Thermo Fischer Scientific, Germany) and 10 mL of *n*-hexane (≥99%, Sigma-Aldrich, Germany) and ground thoroughly in an agate mortar until the powder mixture became dry. The mix was transferred into a 50 mL round-bottom flask and refluxed in 30 mL of *n*-hexane at 80 °C overnight. The powder was isolated by filtration, dried overnight at 70 °C, and calcined at 500 °C for 5 h in a muffle oven. The silica template was removed by two overnight treatments in 2 M NaOH (pellets, ≥98%, puriss. p.a., ACS reagent, Sigma-Aldrich, Germany) at 80 °C. The obtained mesoporous Co_3_O_4_ powder was washed twice with deionized water and once with ethanol and dried at 70 °C overnight.

### Characterization

The nitrogen adsorption–desorption isotherm was recorded at 77 K (−196 °C) using an Anton Paar QuantaTech Inc. Autosorb iQ2 instrument (Boynton Beach, FL, USA). Prior to the measurement, the sample was outgassed overnight at 80 °C under vacuum. Data evaluation was performed using the software provided by the manufacturer (ASiQWin 5.2). The Brunauer–Emmett–Teller (BET) theory was applied on the relative pressure range 0.125–0.275 *P*/*P*_0_ to determine the BET specific surface area (SSA) and the total pore volume *V*_total_ was obtained at *P*/*P*_0_ = 0.95.^65^ The nonlocal density functional theory (NLDFT) kernel for SiO_2_ surfaces and cylindrical pore geometries was used on the adsorption branch of the isotherm to calculate the pore size distribution, NLDFT pore volume and NLDFT SSA. The (metastable) adsorption was favored over the desorption (equilibrium) branch due to the hysteresis loop closing at around *P*/*P*_0_ = 0.45, which could overlap with pore blocking or cavitation effects and, therefore, lead to artifacts.^66^

The low-angle and wide-angle powder X-ray diffraction (PXRD) patterns were obtained with a PANalytical EMPYREAN equipped with a PIXcel3D detector (Malvern PANalytical, United Kingdom), using Cu Kα radiation (45 kV, 40 mA). The Focusing Mirror geometry setup was mounted for the low-angle PXRD measurement (0.5–4 2θ), which was performed in transmission mode with a data acquisition time per step of 50 s. The wide-angle PXRD measurement was recorded from 10 to 90 2θ with Bragg–Brentano HD reflection geometry setup and a data acquisition time per step of 300 s. Both measurements were recorded in continuous mode, employing a step size 2θ of 0.013°.

X-ray photoelectron spectroscopy (XPS) spectra were recorded using a Nexsa (Thermo Scientific, MA, USA) instrument equipped with an Al Kα radiation source (72 W) and an integrated flood gun. The survey spectrum was obtained using a pass energy of 200 eV and an energy step size of 1 eV. The C 1s and Co 2p high-resolution spectra were acquired by measuring 50 passes with a pass energy of 50 eV and an energy step size of 0.1 eV. The deconvolution of charge-corrected spectra (C 1s peak at 284.8 eV) was performed with the Avantage software (Thermo Avantage v5.9922).

### Stability Measurements (Online ICP-MS)

A suspension of ordered mesoporous Co_3_O_4_ (OM-Co_3_O_4_) was prepared with ultrapure water (Milli-Q IQ 7000 Merck) and 2-propanol (Emsure, Merck, ≥99.8% purity) in a ratio of 7:1. Nafion (Sigma-Aldrich, 5 wt %) was added to the suspension as a binder in order to achieve 20 wt % of Nafion in the ink. The suspension was sonicated with the sonication horn (Branson SFX 150) for around 20 min with intervals (4 s pulse, 2 s pause) and 40% intensity until the ink was homogeneous. To prevent the heating of the ink mixture, the vial was kept on ice during sonication. After sonication, the pH of the suspension was adjusted to ∼10 with 1 M KOH before drop-casting 0.25 μL of the suspension on a freshly polished glassy carbon (GC) plate (5 × 5 cm^2^, Sigradur G, HTW), serving as a working electrode. The loading of the catalysts was aimed to be 20–25 μg cm^–2^. The quality and the area of the drop-casted spots (average area of ca. 0.015 cm^2^) were examined with the use of the optical microscope (Keyence VK-X250).

The stability of the drop-casted samples was examined with a scanning flow cell (SFC) combined with inductively coupled plasma mass spectrometry (ICP-MS). A GC rod and an Ag/AgCl electrode (Metrohm) were used as counter and reference electrodes (CE and RE), respectively. Freshly prepared 0.05 M H_2_SO_4_ (96% Suprapur, Merck), saturated with Ar was used as an electrolyte and purged through the setup with a flow rate of 3.6 ± 0.1 μL s^–1^. The electrolyte flow rate was controlled by the peristaltic pump of the ICP-MS (Elemental Scientific M2). The ICP-MS (PerkinElmer NexION 350 × ICP-MS) instrument was calibrated daily with known amounts of analyzed metal (Co^59^) and internal standard (Ge^74^). The working electrode was placed on a translational stage (Physik Instrumente M-403) that allows the SFC to quickly move along the electrode and rapidly screen multiple samples. All electrochemical measurements were performed using a Gamry Reference 600 potentiostat. All instruments (gas control box, mass flow controllers, peristaltic pump, and translational stage) were controlled by homemade LabView software.

### Electrochemical Protocols

Several electrochemical protocols were employed to assess the stability of the catalyst in a wide range potential window and under the OER conditions. First, the so-called accelerated stress test (AST) containing a hold at varied potentials (0.05 and 1.00 *V*_RHE_) followed by 200 cycles of cyclic voltammogram (CV) measurements at a scan rate of 200 mV s^–1^ in a potential range of 1.2 *V*_RHE_ (or 1.4 *V*_RHE_) to 1.7 *V*_RHE_ and finished with the hold at the same potential as in the first step. This protocol was employed to gain general information about the potential stabilization of Co_3_O_4_ under harsh electrochemical conditions at low pH. To have a closer look at the processes occurring in the OER/pre-OER potential range and assess the material’s stability during the power on/off operation, we designed the second protocol: the galvanostatic holds at open circuit potential (OCP) (10 min at the start, 2 min at each following step) and current applied to reach 1 mA cm^–2^ current density (2 min each step) were applied. Third, two CV cycles were measured in the potential range of 0.05 *V*_RHE_ < *E* < 1.8 *V*_RHE_, with a scan rate of 2 mV s^–1^ to follow the dissolution of Co and resolve the anodic and cathodic peaks under constantly changing potential. In order to investigate the potential stability window, we applied the fourth group of the protocols consisting of the stepwise change of potential in the range from 1 to 1.8 *V*_RHE_ in anodic and cathodic directions with the step of 20 or 50 mV and holds at each step of 2–5 min. To ensure reproducibility of the results, all measurements were repeated at least twice on individual pristine drop-cast catalyst spots. Differences in surface areas were accounted for by normalizing the absolute currents with the geometric surface area of the catalyst spots employed as working electrodes.

### DEMS Measurements

Before each measurement, we cleaned all glass and Teflon equipment and platinum wires in a solution of NOCHROMIX (Sigma-Aldrich) in concentrated H_2_SO_4_ (Merck, EMSURE, 98%) overnight. We stored all parts made out of polyetheretherketone in a solution of KMnO_4_ (6.33 mM) in ultrapure water for 1 day. In later case, we removed formed manganese dioxide by diluted piranha solution [H_2_SO_4_ (Emsure 98%) and H_2_O_2_ (Supelco, 30%); 2:1]. Before the experiments, we cleaned the equipment by three cycles of rinsing (5 times) and boiling (30 min) in ultrapure water (Milli-Q Synergy UV, 18.2 MΩ·cm at 25 °C, TOC < 5 ppb). We annealed all noble metal wires in the flame of a Bunsen burner and rinsed these subsequently with ultrapure water. For the measurements in H_2_^18^O, we dried the equipment before use under a nitrogen stream.

Before use, we cleaned an Au substrate (99.999%) in freshly prepared piranha acid [H_2_SO_4_(conc.)/H_2_O_2_(30%) 2:1]. Afterward the crystal was thoroughly rinsed with ultrapure water (Milli-Q synergy UV, 18.2 MΩ cm at 25 °C, <5 ppb TOC) and annealed in the flame of a Bunsen burner at orange glow for 3 min. After the surface was cooled down, we coated it with 10 μL cobalt oxide ink and let it dry for 30 min. The ink was prepared by following the same procedure as described above, except for the amount of dispersed powder OM-Co_3_O_4_ to reach a higher loading. The loading was aimed to be 120 μg cm^–2^.

We measured DEMS using a home-built setup. The setup consists of a commercial inlet microchip (SpectroInlets),^67^ a home build electrochemical cell, a high vacuum compartment, a quadrupole mass spectrometer (Pfeiffer, 220 QMG), and a commercial potentiostat (AutoLab PGSTAT204). We provide further details about the used DEMS setup in our previous publication.^68^

Single mass spectra were recorded with a dwell time of 50 ms per mass. We performed the experiments using the prepared cobalt oxide surface as working electrode and a platinum wire (Hauner 99.999%) as CE. As RE, we used home-built reversible hydrogen electrodes (measurements in H_2_^16^O) or Pd/H electrodes (measurements in H_2_^18^O). To prepare the Pd/H electrodes, we follow a recipe by Vasile and Enke.^69^ In this work, we refer all potentials to the RHE (+50 mV_Pd/H_). The 97% H_2_^18^O water (1 mL) was used to prepare 0.05 M H_2_SO_4_ (Merck, EMSURE, 98%) solution as an electrolyte.

### IL-TEM Measurements

IL-STEM characterization of Co_3_O_4_ was carried out by drop-casting an ink (1 mg mL^–1^ Co_3_O_4_ in Milli-Q water (18.2 MΩ cm) and Nafion solution (D520, Ion Power), added at a ratio of 1:4 between ionomer and Co_3_O_4_), on a gold TEM grid (Agar Scientific, Holey Carbon Films on 300 Mesh Gold). The prepared grid was then assembled into a modified floating apparatus, in detail described in our previous publications,^70^ and used as a working electrode in electrochemical experiments. All measurements were performed with a Biologic SP-300 potentiostat in 0.05 M H_2_SO_4_ (96%, Supelco) solution with a glassy carbon rod and reversible hydrogen electrode (HydroFlex, Gaskatel GmbH) as counter and reference electrodes, respectively. The system was continuously purged with Ar throughout the experiment. Three electrochemical protocols were tested, with two of them starting with five CV between 1.3 and 1.7 V s, measured with a 20 mV s^–1^ scan rate. In the first protocol, a dynamic perturbation, simulating the power on/off conditions, was applied. This was done by 100 repetitions of 2 min galvanostatic hold at 1 mA cm^–2^ and 2 min hold at OCP. The second stationary protocol was continued by a 400 min potential hold at 1.75 V. A third grid was contacted with the electrolyte at OCP for 10 min to test the effect of the contact on the structural stability of Co_3_O_4_.

Before and after electrochemical protocols, TEM characterization was performed on the grids with a JEOL-ARM 200CF, operated at 80 kV. Data was acquired in a STEM mode [bright-field (BF) and HAADF] with a camera length of 8 cm, providing a collection angle range of 68–175 mrad.

## Data Availability

The data that support the findings of this study are presented in the Manuscript and the Supporting Information. Source data are provided at Zenodo: https://doi.org/10.5281/zenodo.14640062.

## Supporting Information Available

Scheme of the spinel structure of Co_3_O_4_; N_2_ physisorption, XRD, and XPS results; dissolution profiles of OM-Co_3_O_4_ and commercial Co_3_O_4_; dissolution profiles of OM-Co_3_O_4_ under varied AST conditions; *S*-number of OM-Co_3_O_4_; Pourbaix diagram of Co_3_O_4_; dissolution profiles of OM-Co_3_O_4_ under varied stepwise protocols; additional DEMS results; HR-IL-TEM images of OM-Co_3_O_4_ before and after the contact with electrolyte; proposed equations to describe the (electro)chemical reactions (PDF)

## References

[ref1] Wang Q. L.; Cheng Y. Q.; Tao H. B.; Liu Y. H.; Ma X. H.; Li D. S.; Yang H. B.; Liu B. Long-Term Stability Challenges and Opportunities in Acidic Oxygen Evolution Electrocatalysis. Angew. Chem., Int. Ed. 2023, 62 (11), e202216645 10.1002/anie.202216645. 36546885

[ref2] Chen F. Y.; Wu Z. Y.; Adler Z.; Wang H. T. Stability challenges of electrocatalytic oxygen evolution reaction: From mechanistic understanding to reactor design. Joule 2021, 5 (7), 1704–1731. 10.1016/j.joule.2021.05.005.

[ref3] Wang Z. B.; Zheng Y. R.; Chorkendorff I.; Norskov J. K. Acid-Stable Oxides for Oxygen Electrocatalysis. ACS Energy Lett. 2020, 5 (9), 2905–2908. 10.1021/acsenergylett.0c01625.

[ref4] Vass A.; Endrodi B.; Samu G. F.; Balog A.; Kormanyos A.; Cherevko S.; Janaky C. Local Chemical Environment Governs Anode Processes in CO_2_ Electrolyzers. ACS Energy Lett. 2021, 6 (11), 3801–3808. 10.1021/acsenergylett.1c01937. 34796265 PMC8593866

[ref5] Wang C.; Lee K.; Liu C. P.; Kulkarni D.; Atanassov P.; Peng X.; Zenyuk I. V. Design of PEM water electrolysers with low iridium loading. Int. Mater. Rev. 2024, 69 (1), 3–18. 10.1177/09506608231216665.

[ref6] Chatti M.; Gardiner J. L.; Fournier M.; Johannessen B.; Williams T.; Gengenbach T. R.; Pai N.; Nguyen C.; MacFarlane D. R.; Hocking R. K.; et al. Intrinsically stable in situ generated electrocatalyst for long-term oxidation of acidic water at up to 80 °C. Nat. Catal. 2019, 2 (5), 457–465. 10.1038/s41929-019-0277-8.

[ref7] Kong S.; Li A. L.; Long J.; Adachi K.; Hashizume D.; Jiang Q. K.; Fushimi K.; Ooka H.; Xiao J. P.; Nakamura R. Acid-stable manganese oxides for proton exchange membrane water electrolysis. Nat. Catal. 2024, 7, 252–261. 10.1038/s41929-023-01091-3.

[ref8] Li A. L.; Ooka H.; Bonnet N.; Hayashi T.; Sun Y. M.; Jiang Q. K.; Li C.; Han H. X.; Nakamura R. Stable Potential Windows for Long-Term Electrocatalysis by Manganese Oxides Under Acidic Conditions. Angew. Chem., Int. Ed. 2019, 58 (15), 5054–5058. 10.1002/anie.201813361. 30869187

[ref9] Cherevko S. Stabilization of non-noble metal electrocatalysts for acidic oxygen evolution reaction. Curr. Opin. Electrochem. 2023, 38, 101213 10.1016/j.coelec.2023.101213.

[ref10] Yang X. L.; Li H. N.; Lu A. Y.; Min S. X.; Idriss Z.; Hedhili M. N.; Huang K. W.; Idriss H.; Li L. J. Highly acid-durable carbon coated Co_3_O_4_ nanoarrays as efficient oxygen evolution electrocatalysts. Nano Energy 2016, 25, 42–50. 10.1016/j.nanoen.2016.04.035.

[ref11] Tran-Phu T.; Chen H. J.; Daiyan R.; Chatti M.; Liu B. R.; Amal R.; Liu Y.; Macfarlane D. R.; Simonov A. N.; Tricoli A. Nanoscale TiO_2_ Coatings Improve the Stability of an Earth-Abundant Cobalt Oxide Catalyst during Acidic Water Oxidation. ACS Appl. Mater. Interfaces 2022, 14 (29), 33130–33140. 10.1021/acsami.2c05849. 35838141

[ref12] Ta X. M. C.; Tran-Phú T.; Yuwono J. A.; Nguyen T. K. A.; Bui A. D.; Truong T. N.; Chang L. C.; Magnano E.; Daiyan R.; Simonov A. N.; et al. Optimal Coatings of Co_3_O_4_ Anodes for Acidic Water Electrooxidation. Small 2024, 20, 2304650 10.1002/smll.202304650. 37863809

[ref13] Yeh Y. X.; Cheng C. C.; Jhu P. S.; Lin S. H.; Chen P. W.; Lu S. Y. Core-shell FTO@Co_3_O_4_ nanoparticles as active and stable anode catalysts for acidic oxygen evolution reaction and proton exchange membrane water electrolysis. J. Mater. Chem. A 2023, 11 (7), 3399–3407. 10.1039/D2TA08541K.

[ref14] Frydendal R.; Paoli E. A.; Chorkendorff I.; Rossmeisl J.; Stephens I. E. L. Toward an Active and Stable Catalyst for Oxygen Evolution in Acidic Media: Ti-Stabilized MnO_2_. Adv. Energy Mater. 2015, 5 (22), 1500991 10.1002/aenm.201500991.

[ref15] Huynh M.; Ozel T.; Liu C.; Lau E. C.; Nocera D. G. Design of template-stabilized active and earth-abundant oxygen evolution catalysts in acid. Chem. Sci. 2017, 8 (7), 4779–4794. 10.1039/C7SC01239J. 29163926 PMC5637126

[ref16] Moreno-Hernandez I. A.; MacFarland C. A.; Read C. G.; Papadantonakis K. M.; Brunschwig B. S.; Lewis N. S. Crystalline nickel manganese antimonate as a stable water-oxidation catalyst in aqueous 1.0 M H_2_SO_4_. Energy Environ. Sci. 2017, 10 (10), 2103–2108. 10.1039/C7EE01486D.

[ref17] Zhou L.; Shinde A.; Montoya J. H.; Singh A.; Gul S.; Yano J.; Ye Y. F.; Crumlin E. J.; Richter M. H.; Cooper J. K.; et al. Rutile Alloys in the Mn-Sb-O System Stabilize Mn^3+^ to Enable Oxygen Evolution in Strong Acid. ACS Catal. 2018, 8 (12), 10938–10948. 10.1021/acscatal.8b02689.

[ref18] Kwong W. L.; Lee C. C.; Shchukarev A.; Messinger J. Cobalt- doped hematite thin films for electrocatalytic water oxidation in highly acidic media. Chem. Commun. 2019, 55 (34), 5017–5020. 10.1039/C9CC01369E. 30968887

[ref19] Etzi Coller Pascuzzi M.; van Velzen M.; Hofmann J. P.; Hensen E. J. M. On the Stability of Co_3_O_4_ Oxygen Evolution Electrocatalysts in Acid. ChemCatChem 2021, 13 (1), 459–467. 10.1002/cctc.202001428.

[ref20] Luke S.; Chatti M.; Yadav A.; Kerr B. V.; Kangsabanik J.; Williams T.; Cherepanov P. V.; Johannessen B.; Tanksale A.; MacFarlane D. R.; et al. Mixed metal-antimony oxide nanocomposites: low pH water oxidation electrocatalysts with outstanding durability at ambient and elevated temperatures. J. Mater. Chem. A 2021, 9 (48), 27468–27484. 10.1039/D1TA07293E.

[ref21] Li A. L.; Kong S.; Guo C. X.; Ooka H.; Adachi K.; Hashizume D.; Jiang Q. K.; Han H. X.; Xiao J. P.; Nakamura R. Enhancing the stability of cobalt spinel oxide towards sustainable oxygen evolution in acid. Nat. Catal. 2022, 5 (2), 109–118. 10.1038/s41929-021-00732-9.

[ref22] Pan S. J.; Li H.; Liu D.; Huang R.; Pan X. L.; Ren D.; Li J.; Shakouri M.; Zhang Q. X.; Wang M. J.; et al. Efficient and stable noble-metal-free catalyst for acidic water oxidation. Nat. Commun. 2022, 13 (1), 2294 10.1038/s41467-022-30064-6. 35484271 PMC9050677

[ref23] Chong L. A.; Gao G. P.; Wen J. G.; Li H. X.; Xu H. P.; Green Z.; Sugar J. D.; Kropf A. J.; Xu W. Q.; Lin X. M.; et al. La- and Mn-doped cobalt spinel oxygen evolution catalyst for proton exchange membrane electrolysis. Science 2023, 380 (6645), 609–616. 10.1126/science.ade1499. 37167381

[ref24] Xie J. Y.; Wang F. L.; Zhai X. J.; Li X.; Zhang Y. S.; Fan R. Y.; Lv R. Q.; Chai Y. M.; Dong B. Manganese doped hollow cobalt oxide catalysts for highly efficient oxygen evolution in wide pH range. Chem. Eng. J. 2024, 482, 148926 10.1016/j.cej.2024.148926.

[ref25] Ram R.; Xia L.; Benzidi H.; Guha A.; Golovanova V.; Garzon Manjon A.; Llorens Rauret D.; Sanz Berman P.; Dimitropoulos M.; Mundet B.; et al. Water-hydroxide trapping in cobalt tungstate for proton exchange membrane water electrolysis. Science 2024, 384 (6702), 1373–1380. 10.1126/science.adk9849. 38900890

[ref26] Sun L.; Feng M.; Peng Y.; Zhao X.; Shao Y. Q.; Yue X.; Huang S. M. Constructing oxygen vacancies by doping Mo into spinel Co_3_O_4_ to trigger a fast oxide path mechanism for acidic oxygen evolution reaction. J. Mater. Chem. A 2024, 12 (15), 8796–8804. 10.1039/D4TA00655K.

[ref27] Huang J. Z.; Sheng H. Y.; Ross R. D.; Han J. C.; Wang X. J.; Song B.; Jin S. Modifying redox properties and local bonding of Co_3_O_4_ by CeO_2_ enhances oxygen evolution catalysis in acid. Nat. Commun. 2021, 12 (1), 3036 10.1038/s41467-021-23390-8. 34031417 PMC8144612

[ref28] Du H. L.; Chatti M.; Kerr B.; Nguyen C. K.; Tran-Phu T.; Hoogeveen D. A.; Cherepanov P. V.; Chesman A. S. R.; Johannessen B.; Tricoli A.; et al. Durable Electrooxidation of Acidic Water Catalysed by a Cobalt-Bismuth-based Oxide Composite: An Unexpected Role of the F-doped SnO_2_ Substrate. ChemCatChem 2022, 14 (11), e202200013 10.1002/cctc.202200013.

[ref29] Zhu S. C.; Yang R. O.; Li H. J. W.; Huang S. R.; Wang H. Z.; Liu Y. W.; Li H. Q.; Zhai T. Y. Reconstructing Hydrogen-Bond Network for Efficient Acidic Oxygen Evolution. Angew. Chem., Int. Ed. 2024, 63, e202319462 10.1002/anie.202319462. 38286750

[ref30] Pastor E.; Lian Z.; Xia L.; Ecija D.; Galan-Mascaros J. R.; Barja S.; Gimenez S.; Arbiol J.; Lopez N.; García de Arquer F. P. Complementary probes for the electrochemical interface. Nat. Rev. Chem. 2024, 8 (3), 159–178. 10.1038/s41570-024-00575-5. 38388837

[ref31] Kasian O.; Geiger S.; Li T.; Grote J. P.; Schweinar K.; Zhang S. Y.; Scheu C.; Raabe D.; Cherevko S.; Gault B.; et al. Degradation of iridium oxides via oxygen evolution from the lattice: correlating atomic scale structure with reaction mechanisms. Energy Environ. Sci. 2019, 12 (12), 3548–3555. 10.1039/C9EE01872G.

[ref32] Natarajan K.; Munirathinam E.; Yang T. C. K. Operando Investigation of Structural and Chemical Origin of Co_3_O_4_ Stability in Acid under Oxygen Evolution Reaction. ACS Appl. Mater. Interfaces 2021, 13 (23), 27140–27148. 10.1021/acsami.1c07267. 34096278

[ref33] Priamushko T.; Guillet-Nicolas R.; Yu M. Q.; Doyle M.; Weidenthaler C.; Tüysüz H.; Kleitz F. Nanocast Mixed Ni-Co-Mn Oxides with Controlled Surface and Pore Structure for Electrochemical Oxygen Evolution Reaction. ACS Appl. Energy Mater. 2020, 3 (6), 5597–5609. 10.1021/acsaem.0c00544.

[ref34] Deeloed W.; Priamushko T.; Cizek J.; Suramitr S.; Kleitz F. Defect-Engineered Hydroxylated Mesoporous Spinel Oxides as Bifunctional Electrocatalysts for Oxygen Reduction and Evolution Reactions. ACS Appl. Mater. Interfaces 2022, 14 (20), 23307–23321. 10.1021/acsami.2c00254. 35561262 PMC9136850

[ref35] Priamushko T.; Budiyanto E.; Eshraghi N.; Weidenthaler C.; Kahr J.; Jahn M.; Tüysüz H.; Kleitz F. Incorporation of Cu/Ni in Ordered Mesoporous Co-Based Spinels to Facilitate Oxygen Evolution and Reduction Reactions in Alkaline Media and Aprotic Li-O_2_ Batteries. ChemSusChem 2022, 15 (5), e202102404 10.1002/cssc.202102404. 34905292 PMC9303656

[ref36] Rumplecker A.; Kleitz F.; Salabas E. L.; Schüth F. Hard templating pathways for the synthesis of nanostructured porous Co_3_O_4_. Chem. Mater. 2007, 19 (3), 485–496. 10.1021/cm0610635.

[ref37] Topalov A. A.; Cherevko S.; Zeradjanin A. R.; Meier J. C.; Katsounaros I.; Mayrhofer K. J. J. Towards a comprehensive understanding of platinum dissolution in acidic media. Chem. Sci. 2014, 5, 631–638. 10.1039/c3sc52411f.

[ref38] Cherevko S. Electrochemical dissolution of noble metals native oxides. J. Electroanal. Chem. 2017, 787, 11–13. 10.1016/j.jelechem.2017.01.029.

[ref39] Zhao J. W.; Yue K. H.; Zhang H.; Wei S. Y.; Zhu J. W.; Wang D. D.; Chen J. Z.; Fominski V. Y.; Li G. R. The formation of unsaturated IrO_x_ in SrIrO_3_ by cobalt-doping for acidic oxygen evolution reaction. Nat. Commun. 2024, 15 (1), 2928 10.1038/s41467-024-46801-y. 38575606 PMC10995174

[ref40] Cherevko S.; Topalov A. A.; Zeradjanin A. R.; Katsounaros I.; Mayrhofer K. J. J. Gold dissolution: towards understanding of noble metal corrosion. RSC Adv. 2013, 3 (37), 16516 10.1039/c3ra42684j.

[ref41] Zlatar M.; Nater D.; Escalera-Lopez D.; Joy R. M.; Pobedinskas P.; Haenen K.; Coperet C.; Cherevko S. Evaluating the stability of Ir single atom and Ru atomic cluster oxygen evolution reaction electrocatalysts. Electrochim. Acta 2023, 444, 141982 10.1016/j.electacta.2023.141982.

[ref42] Van Pham C.; Bühler M.; Knöppel J.; Bierling M.; Seeberger D.; Escalera-López D.; Mayrhofer K. J. J.; Cherevko S.; Thiele S. IrO_2_ coated TiO_2_ core-shell microparticles advance performance of low loading proton exchange membrane water electrolyzers. Appl. Catal., B 2020, 269, 118762 10.1016/j.apcatb.2020.118762.

[ref43] Hoffmeister D.; Finger S.; Fiedler L.; Ma T. C.; Körner A.; Zlatar M.; Fritsch B.; Bodnar K. W.; Carl S.; Götz A.; et al. Photodeposition-Based Synthesis of TiO_2_@IrO_x_ Core-Shell Catalyst for Proton Exchange Membrane Water Electrolysis with Low Iridium Loading. Adv. Sci. 2024, 11, 2402991 10.1002/advs.202402991. PMC11321668 38874424

[ref44] Zhu W. J.; Yao F.; Cheng K. J.; Zhao M. T.; Yang C. J.; Dong C. L.; Hong Q. M.; Jiang Q.; Wang Z. C.; Liang H. F. Direct Dioxygen Radical Coupling Driven by Octahedral Ruthenium-Oxygen-Cobalt Collaborative Coordination for Acidic Oxygen Evolution Reaction. J. Am. Chem. Soc. 2023, 145 (32), 17995–18006. 10.1021/jacs.3c05556. 37550082

[ref45] Lee K.; Shim J.; Ji H.; Kim J.; Lee H. S.; Shin H.; Bootharaju M. S.; Lee K. S.; Ko W.; Lee J.; et al. Tailoring cobalt spinel oxide with site-specific single atom incorporation for high-performance electrocatalysis. Energy Environ. Sci. 2024, 17 (10), 3618–3628. 10.1039/D4EE00058G.

[ref46] Geiger S.; Kasian O.; Ledendecker M.; Pizzutilo E.; Mingers A. M.; Fu W. T.; Diaz-Morales O.; Li Z. Z.; Oellers T.; Fruchter L.; et al. The stability number as a metric for electrocatalyst stability benchmarking. Nat. Catal. 2018, 1 (7), 508–515. 10.1038/s41929-018-0085-6.

[ref47] Daiane Ferreira da Silva C.; Claudel F.; Martin V.; Chattot R.; Abbou S.; Kumar K.; Jiménez-Morales I.; Cavaliere S.; Jones D.; Rozière J.; et al. Oxygen Evolution Reaction Activity and Stability Benchmarks for Supported and Unsupported IrO_x_ Electrocatalysts. ACS Catal. 2021, 11 (7), 4107–4116. 10.1021/acscatal.0c04613.

[ref48] Wang Z. B.; Guo X. Y.; Montoya J.; Norskov J. K. Predicting aqueous stability of solid with computed Pourbaix diagram using SCAN functional. npj Comput. Mater. 2020, 6 (1), 160 10.1038/s41524-020-00430-3.

[ref49] Haase F. T.; Bergmann A.; Jones T. E.; Timoshenko J.; Herzog A.; Jeon H. S.; Rettenmaier C.; Cuenya B. R. Size effects and active state formation of cobalt oxide nanoparticles during the oxygen evolution reaction. Nat. Energy 2022, 7 (8), 765–773. 10.1038/s41560-022-01083-w.

[ref50] Binninger T.; Mohamed R.; Waltar K.; Fabbri E.; Levecque P.; Kötz R.; Schmidt T. J. Thermodynamic explanation of the universal correlation between oxygen evolution activity and corrosion of oxide catalysts. Sci. Rep. 2015, 5, 12167 10.1038/srep12167. 26178185 PMC4503990

[ref51] Grimaud A.; Diaz-Morales O.; Han B. H.; Hong W. T.; Lee Y. L.; Giordano L.; Stoerzinger K. A.; Koper M. T. M.; Shao-Horn Y. Activating lattice oxygen redox reactions in metal oxides to catalyse oxygen evolution. Nat. Chem. 2017, 9 (8), 457–465. 10.1038/nchem.2695. 28430191

[ref52] Kasian O.; Grote J. P.; Geiger S.; Cherevko S.; Mayrhofer K. J. J. The Common Intermediates of Oxygen Evolution and Dissolution Reactions during Water Electrolysis on Iridium. Angew. Chem., Int. Ed. 2018, 57 (9), 2488–2491. 10.1002/anie.201709652. PMC5838529 29219237

[ref53] Wang L.; Wen X.; Lai X. J.; Shi H. Q.; Li Y.; Wang C. Nanometer-thick iridium oxide layer coated spinel cobalt oxide nanoparticles for electrocatalytic oxygen evolution in acid. Int. J. Hydrogen Energy 2024, 78, 1192–1200. 10.1016/j.ijhydene.2024.06.366.

[ref54] Wang G. W.; Zhang G. K.; Chen X. Ru Single Atoms Integrated into Cobalt Oxide Spinel Structure with Interstitial Carbon for Enhanced Electrocatalytic Water Oxidation. Small 2024, 20, 2310372 10.1002/smll.202310372. 38196048

[ref55] Bergmann A.; Martinez-Moreno E.; Teschner D.; Chernev P.; Gliech M.; de Araújo J. F.; Reier T.; Dau H.; Strasser P. Reversible amorphization and the catalytically active state of crystalline Co_3_O_4_ during oxygen evolution. Nat. Commun. 2015, 6, 8625 10.1038/ncomms9625. 26456525 PMC4633955

[ref56] Bouvier M.; Bubi I. P.; Wiegmann T.; Qiu C. R.; Allongue P.; Magnussen O. M.; Maroun F. Unraveling the Cobalt Oxidation State at the Surface of Epitaxial Cobalt Oxide Films during the Oxygen Evolution Reaction by X-ray Absorption Spectroscopy/Surface X-ray Diffraction. ACS Appl. Energy Mater. 2023, 6 (14), 7335–7345. 10.1021/acsaem.3c00211.

[ref57] Pan S. J.; Li H.; Wang T. Y.; Fu Y.; Wang S. N.; Xie Z. S.; Wei L.; Li H.; Li N. Er-Doping Enhances the Oxygen Evolution Performance of Cobalt Oxide in Acidic Medium. ACS Catal. 2024, 14 (18), 13814–13824. 10.1021/acscatal.4c03088. PMC12053946 40337370

[ref58] Huang J.; Borca C. N.; Huthwelker T.; Yüzbasi N. S.; Baster D.; El Kazzi M.; Schneider C. W.; Schmidt T. J.; Fabbri E. Surface oxidation/spin state determines oxygen evolution reaction activity of cobalt-based catalysts in acidic environment. Nat. Commun. 2024, 15 (1), 3067 10.1038/s41467-024-47409-y. 38594282 PMC11003995

[ref59] Sheng Z. Y.; Wang S. H.; Jiang Q.; Ni Y. M.; Zhang C. R.; Ahmad A.; Song F. Crystal facet evolution of spinel Co_3_O_4_ nanosheets in acidic oxygen evolution catalysis. Catal. Sci. Technol. 2023, 13 (15), 4542–4549. 10.1039/D3CY00713H.

[ref60] Simondson D.; Chatti M.; Bonke S. A.; Tesch M. F.; Golnak R.; Xiao J.; Hoogeveen D. A.; Cherepanov P. V.; Gardiner J. L.; Tricoli A.; et al. Stable Acidic Water Oxidation with a Cobalt-Iron-Lead Oxide Catalyst Operating via a Cobalt-Selective Self-Healing Mechanism. Angew. Chem., Int. Ed. 2021, 60 (29), 15821–15826. 10.1002/anie.202104123. 33884730

[ref61] Li L.; Cao X. J.; Huo J. J.; Qu J. P.; Chen W. H.; Liu C. T.; Zhao Y. F.; Liu H.; Wang G. X. High valence metals engineering strategies of Fe/Co/Ni-based catalysts for boosted OER electrocatalysis. J. Energy Chem. 2023, 76, 195–213. 10.1016/j.jechem.2022.09.022.

[ref62] Han W. W.; Qian Y.; Zhang F.; He Y.; Li P.; Zhang X. W. Ultrasmall IrO_2_ nanoparticles anchored on hollow Co-Mo multi-oxide heterostructure nanocages for efficient oxygen evolution in acid. Chem. Eng. J. 2023, 473, 145353 10.1016/j.cej.2023.145353.

[ref63] Kleitz F.; Choi S. H.; Ryoo R. Cubic Ia3d large mesoporous silica:: synthesis and replication to platinum nanowires, carbon nanorods and carbon nanotubes. Chem. Commun. 2003, 46 (17), 2136–2137. 10.1039/b306504a. 13678168

[ref64] Yen H.; Seo Y.; Guillet-Nicolas R.; Kaliaguine S.; Kleitz F. One-step-impregnation hard templating synthesis of high-surface-area nanostructured mixed metal oxides (NiFe_2_O_4_, CuFe_2_O_4_ and Cu/CeO_2_). Chem. Commun. 2011, 47 (37), 10473–10475. 10.1039/c1cc13867g. 21858306

[ref65] Thommes M.; Kaneko K.; Neimark A. V.; Olivier J. P.; Rodriguez-Reinoso F.; Rouquerol J.; Sing K. S. W. Physisorption of gases, with special reference to the evaluation of surface area and pore size distribution (IUPAC Technical Report). Pure Appl. Chem. 2015, 87 (9–10), 1051–1069. 10.1515/pac-2014-1117.

[ref66] Schlumberger C.; Thommes M. Characterization of Hierarchically Ordered Porous Materials by Physisorption and Mercury Porosimetry-A Tutorial Review. Adv. Mater. Interfaces 2021, 8 (4), 2002181 10.1002/admi.202002181.

[ref67] Trimarco D. B.; Pedersen T.; Hansen O.; Chorkendorff I.; Vesborg P. C. K. Fast and sensitive method for detecting volatile species in liquids. Rev. Sci. Instrum. 2015, 86 (7), 075006 10.1063/1.4923453. 26233407

[ref68] Stumm C.; Kastenmeier M.; Waidhas F.; Bertram M.; Sandbeck D. J. S.; Bochmann S.; Mayrhofer K. J. J.; Bachmann J.; Cherevko S.; Brummel O.; et al. Model electrocatalysts for the oxidation of rechargeable electrofuels - carbon supported Pt nanoparticles prepared in UHV. Electrochim. Acta 2021, 389, 138716 10.1016/j.electacta.2021.138716.

[ref69] Vasile M. J.; Enke C. G. Preparation and Thermodynamic Properties of a Palladium-Hydrogen Electrode. J. Electrochem. Soc. 1965, 112 (8), 865 10.1149/1.2423713.

[ref70] Hrnjic A.; Ruiz-Zepeda F.; Gaberscek M.; Bele M.; Suhadolnik L.; Hodnik N.; Jovanovic P. Modified Floating Electrode Apparatus for Advanced Characterization of Oxygen Reduction Reaction Electrocatalysts. J. Electrochem. Soc. 2020, 167 (16), 166501 10.1149/1945-7111/abc9de.

