# Supplementary material for: Be Aware of Transient Dissolution Processes in Co3O4 Acidic Oxygen Evolution Reaction Electrocatalysts
Source: J Am Chem Soc. 2025 Jan 15;147(4):3517–28. doi: 10.1021/jacs.4c14952 (PMC11783546; doi:10.1021/jacs.4c14952)
Supplement: Supplementary file 1 — ja4c14952_si_001.pdf [file ja4c14952_si_001.pdf]

Supporting Information

Be Aware of Transient Dissolution Processes in Co_3O_4 Acidic Oxygen Evolution Reaction Electrocatalysts

Tatiana Priamushko[1,], Evanie Franz[2], Anja Logar[3,4], Lazar Bijelić[3,4], Patrick Guggenberger[5,6], Daniel Escalera-López[1], Matej Zlatar[1], Jörg Libuda[2], Freddy Kleitz[5], Nejc Hodnik[3,4], Olaf Brummel[2], Serhiy Cherevko[1,*]*

[1] Helmholtz-Institute Erlangen-Nürnberg for Renewable Energy (IET-2), Forschungszentrum Jülich, 91058 Erlangen, Germany

[2] Interface Research and Catalysis, ECRC, Friedrich-Alexander-Universität Erlangen-Nürnberg, 91058 Erlangen, Germany

[3] Department of Materials Chemistry, National Institute of Chemistry, 1000 Ljubljana, Slovenia

[4] University of Nova Gorica, Vipavska 13, 5000 Nova Gorica, Slovenia

[5] Department of Functional Materials and Catalysis, University of Vienna, 1090 Vienna, Austria

[6] Vienna Doctoral School in Chemistry (DoSChem), University of Vienna, 1090 Vienna, Austria

Corresponding authors: t.priamushko@fz-juelich.de; s.cherevko@fz-juelich.de.

Chapter 1. Physicochemical properties.

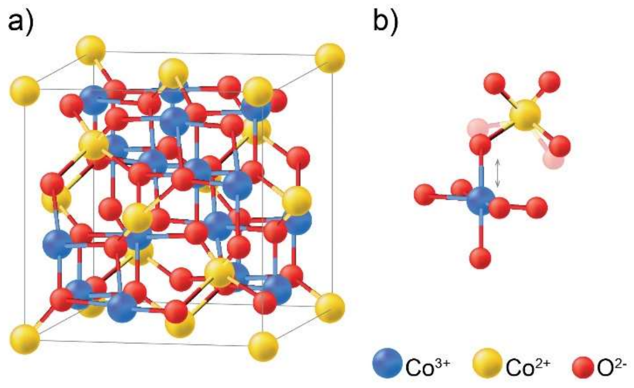

Figure S1. Schematic representation of the spinel structure of Co_3O_4.

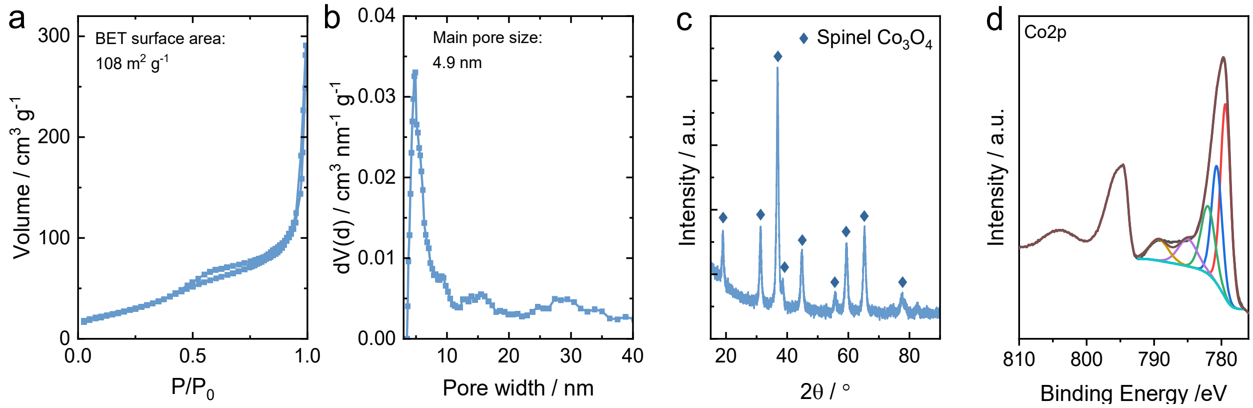

Figure S2. N_2 physisorption isotherm (a), pore size distribution (PSD) (b), XRD pattern (c), and XPS spectrum of Co2p (d) obtained for the OM-Co_3O_4 in this work.

Figure S2a depicts a typical for such mesoporous replicas type IVa isotherm with a hysteresis loop.[1] Non-local density functional theory (NLDFT)[1, 2] pore size analysis suggests a narrow pore size distribution (PSD) (Figure S2b) with most pores being 4.9 nm in size. The error of the NLDFT fitting in this case is 1.36%.

Figure S2c demonstrates that OM-Co_3O_4 exhibits a pure spinel phase according to the obtained X-ray diffraction (XRD) pattern. Figure S2d depicts Co 2p spectrum obtained by X-ray photoelectron spectroscopy (XPS). The Co $2p_{3/2}$ high-resolution (HR) XPS spectrum was deconvoluted using

the fitting procedure for Co_3O_4 described by Biesinger et al.[3] The peaks are compared to the Co_3O_4 spectrum presented in the literature. The satellite peak at 785.0 eV displays a slightly higher intensity, which hints on the presence of trace amounts of $Co(OH)_2$ at the surface of the OM-Co_3O_4. The latter could result from the harsh 2M NaOH template removal step at 80 °C.[4] This slight segregation of Co^{II} species on the surface of the material might affect the contact dissolution in our study.

Chapter 2. Dissolution of Co_3O_4.

To ensure that the behavior of the OM-Co_3O_4 is comparable to the one of the commercial Co_3O_4 (c-Co_3O_4), we ran electrochemical online ICP-MS measurements. The obtained results (Figure S3) demonstrate that OM-Co_3O_4 behaves identically to the c-Co_3O_4. Higher dissolution of the mesoporous oxide can be assigned to a higher surface area of the porous material.

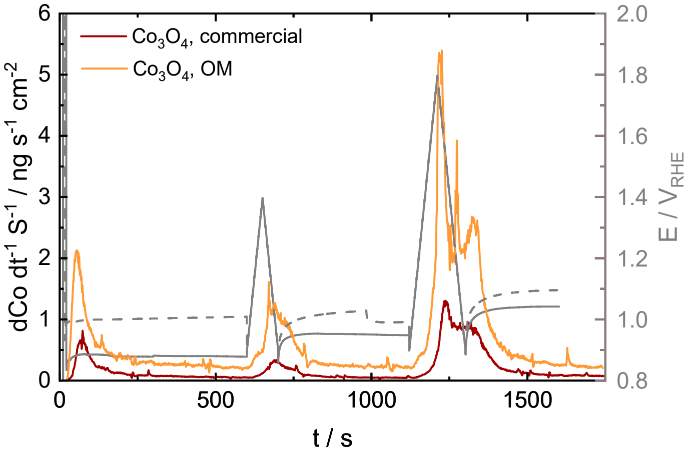

Figure S3. Comparison of dissolution profiles of OM-Co_3O_4 and commercial Co_3O_4 in 0.1M $HClO_4$. The loading of the samples was similar and in the range of 20-25 µg cm^{-2}. The materials were contacted at OCP and then kept at these conditions for 10 min, after which the CV from 0.9 to 1.4 V_{RHE} was recorded at 10 mV s^{-1}. This step was followed by the second OCP hold for 7 min and further CV from 0.9 to 1.8 V_{RHE}. The final step was the third OCP hold for 5 min. The grey lines depict the change in potential (E / V_{RHE}).

These measurements were performed in 0.1M $HClO_4$ in order to compare the behavior of various cobalt oxides. However, all the further experiments were performed in 0.05M H_2SO_4 to ensure an easier comparison with the data published in the literature.

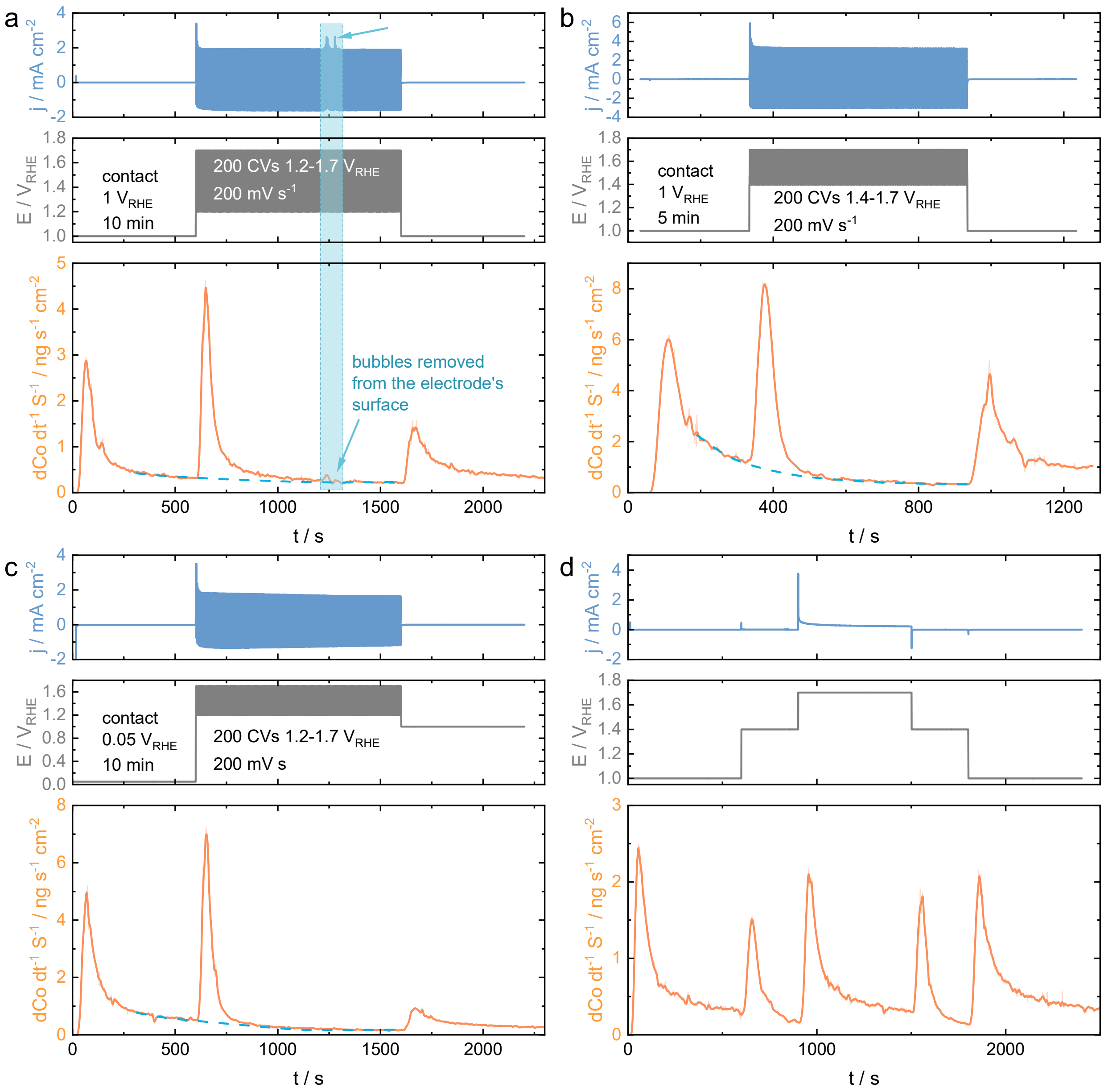

Figure S4. Dissolution profiles of Co under varied AST protocol conditions (a-c) and potentiostatic holds (d). Blue curves represent the collected current density (data normalized by the geometric surface area of the electrode), grey curves represent the potential applied during the protocol, and the orange curves represent the dissolution profile of Co. The dashed blue line in Figures (a-c) presents a suggested dissolution profile of cobalt if the condition did not change and no electrochemical protocol was applied after the contact of the electrode and the electrolyte.

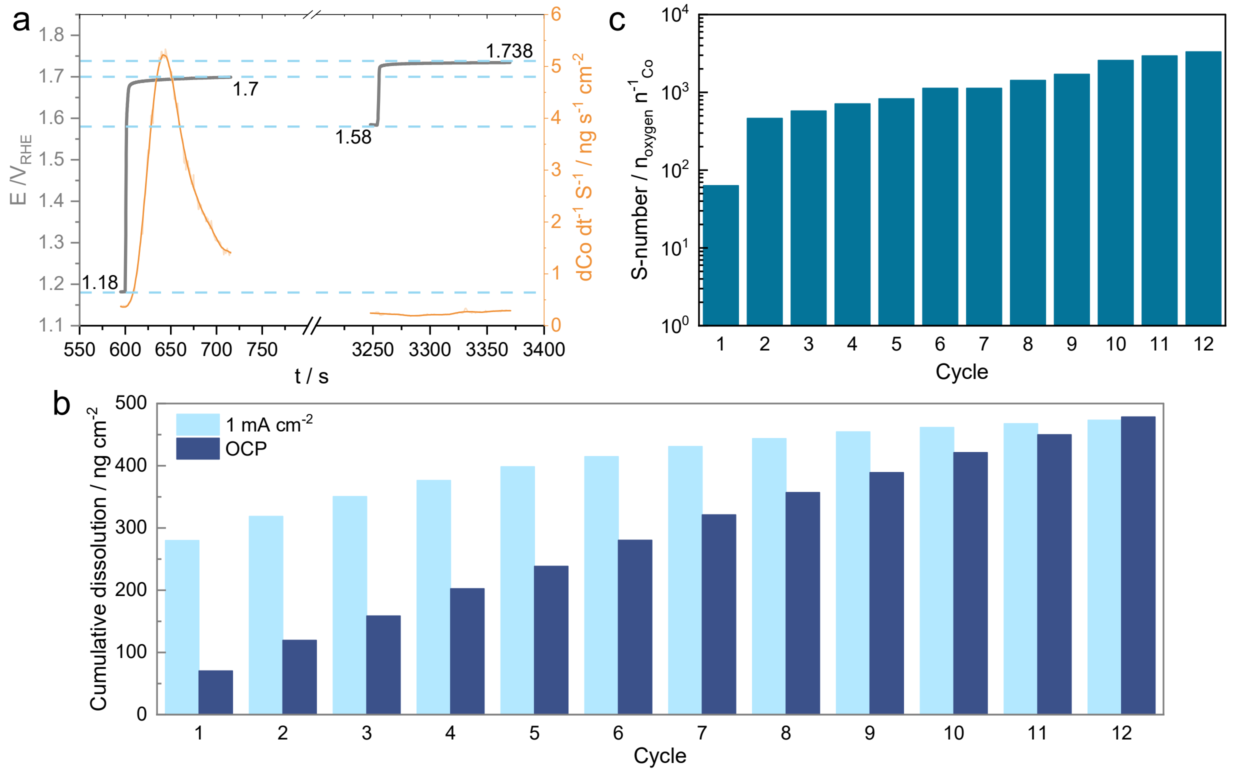

Figure S5. A comparison between the potential and dissolution rates during the first and the last anodic holds (a), the cumulative dissolution (b), and the stability number (S-number) of cobalt (c) calculated from the experiment presented in Figure 1b.

As seen in Figure S5a, the change in the potential during the first anodic hold is substantially larger than during the last one. Therefore, it is not surprising that we observe a much higher dissolution rate at the start of the first hold and almost no dissolution at the last one. Moreover, one can notice that the decrease in dissolution rate during the first anodic hold overlaps with the potential reaching a relatively constant value. The potential during the last hold reaches a constant value almost from the start, which might also contribute to the low dissolution rate of cobalt.

Assuming that the dissolution of cobalt is transient and is induced by the redox reactions (assumingly Co^{III} to $Co^{III\text{-}IV}$ redox couple) during this protocol, one can compare the dissolved amounts of cobalt ions during the anodic and OCP holds. Figure S5b presents the cumulative dissolution during all the subsequent anodic and OCP holds. Rapid initial surface oxidation results in high dissolution of cobalt, as was discussed above. The reduction, on the other hand, seems to be slower and steadily leads to similar dissolution rates regardless of the cycle number. While the oxidation cumulative dissolution almost reaches the plateau within 10 cycles, the cumulative

dissolution due to the reduction of Co^{III-IV}-based species exhibits a linear growth within the number of cycles. All this brings us to the following assumptions: (a) the process of the formation of the Co^{III-IV}-based species induces lower dissolution than the process of their reduction, and (b) formed Co^{III-IV}-based species are stable, and the surface of Co_3O_4 is getting passivated when these species are formed as the dissolution during the hold is not steady but decreases rapidly.

Figure S5c compares the stability number calculated from each anodic hold of this protocol. This is the most convincing proof of the Co_3O_4 stabilization with each subsequent hold as the S-number is increasing and reaching the value of 3325.

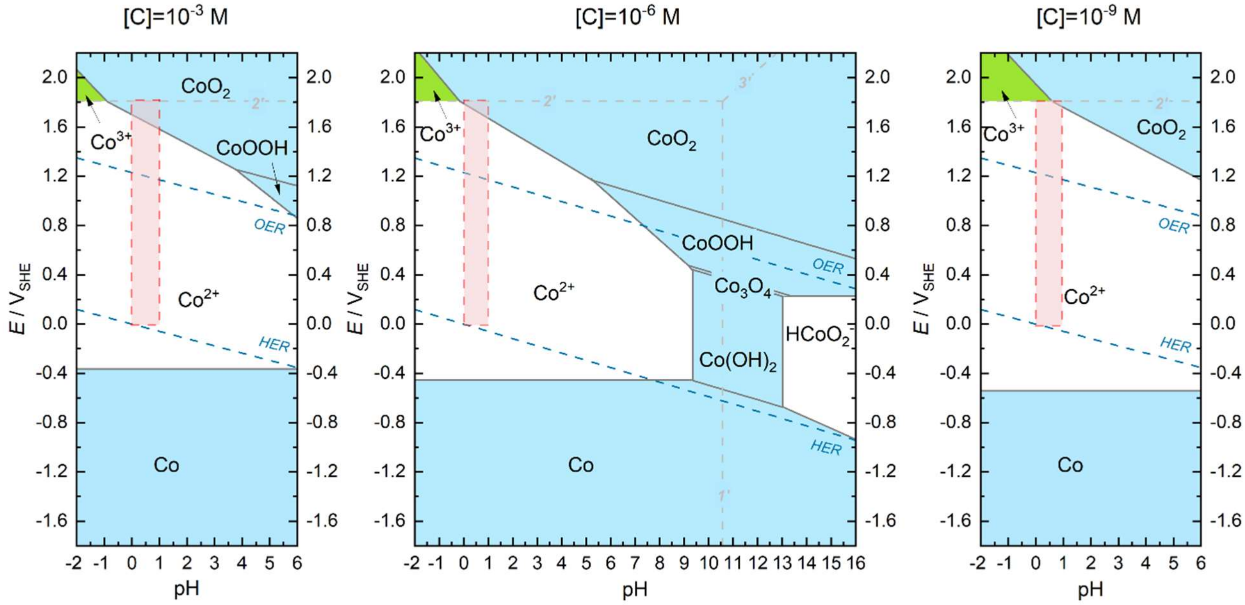

Figure S6. Pourbaix diagrams of cobalt calculated from experimental thermodynamic tables with aqueous ion concentrations 10^{-3} M (left), 10^{-6} M (center), and 10^{-9} M (right) at 25 °C. To avoid the extra complexity of the graph, the diagram for the lower and higher ion concentrations were cut to show only lower pH regions. The red area indicates the potential range and pH values at which all the experiments were performed in this work. The blue areas indicate the solid stable species areas, while the white ones indicate corrosion regions (dissolved species). The green area indicates the region of Co^{3+} ions. The faint dashed lines represent the limits of the domains of the relative predominance of the dissolved substances according to the following data:

1′: Co^{2+} / $HCoO_2^-$ *pH=10.57*

2′: Co^{2+} / Co^{3+} *E_0=1.808*

3′: $HCoO^-{}_2$ / Co^{3+} *E_0=-0.065+0.1773pH*

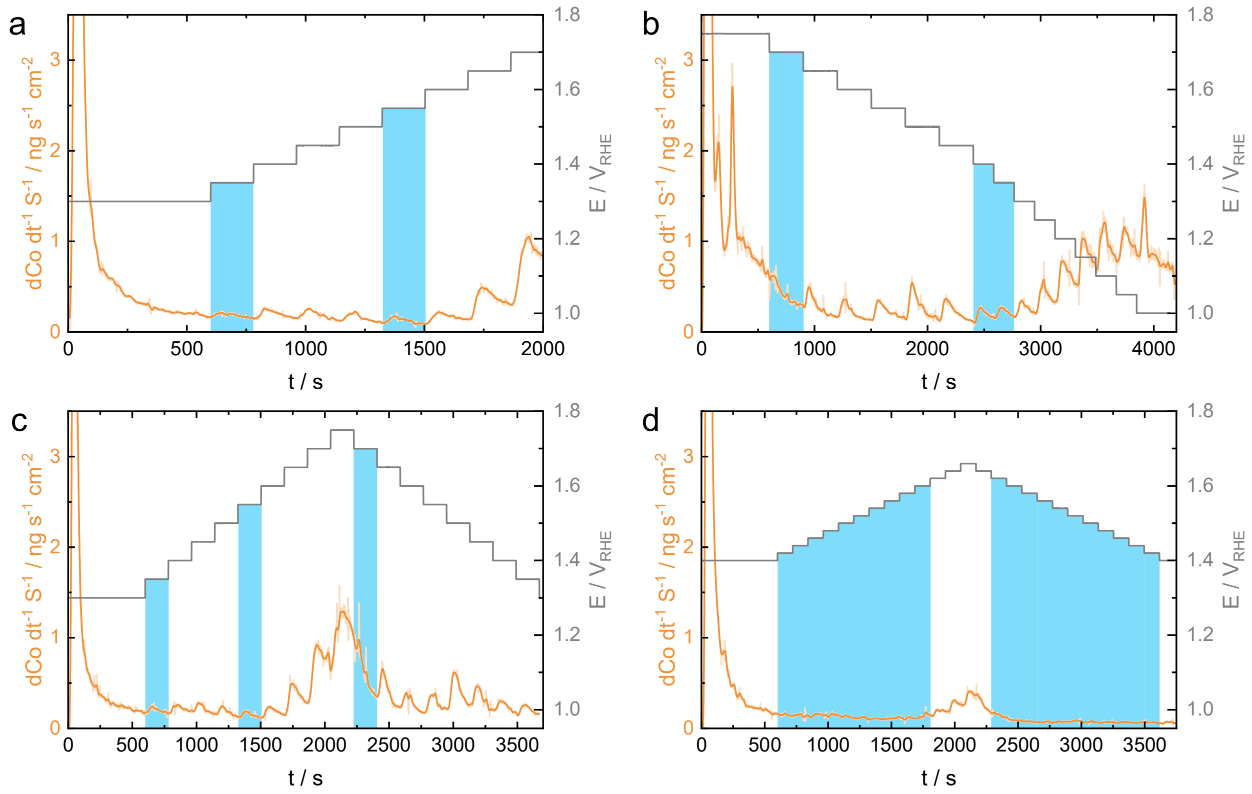

Figure S7. Dissolution profiles of Co under varied stepwise protocols: (a) anodic steps of 50 mV from 1.30 V_{RHE} to 1.7 V_{RHE}, (b) cathodic steps of 50 mV from 1.75 V_{RHE} to 1.00 V_{RHE}, (c) both directions with step of 50 mV from 1.30 V_{RHE} to 1.75 V_{RHE}, and (d) both directions from 1.40 V_{RHE} to 1.66 V_{RHE} with steps of 20 mV. Potential regions with the lowest dissolution are marked in blue.

Several electrochemical protocols with various potential steps and potential windows are presented here in Figure S7. Anodically changing potential with the step of 50 mV does not induce high dissolution of cobalt, except for the potentials higher than 1.60 V_{RHE} (Figure S7a). When the potential changes stepwise in the cathodic direction (Figure S7b), overall higher dissolution is initiated at every step. Especially high dissolution rates are observed for the potentials lower than 1.30 V_{RHE}, which correlates with the intense dissolution peaks observed during the CV measurements (Figure 2a-b). Figure S7c presents a combined protocol, where the potential changes in a stepwise manner in both anodic and cathodic directions. The highest dissolution rates occur at the OER potential (1.65 V_{RHE} and higher). Once again, changes in the potential in the cathodic direction cause higher dissolution than when it changes in the anodic direction.

Importantly, the dissolution of cobalt oxide can be minimized by changing the conditions, e.g., potential step and window, as shown in Figure S7d. Here, a very narrow potential window (the stabilization region) was studied. As we observed from the CV experiments, a stabilization of the catalyst should be observed in this potential window. However, when the potential step was higher (here, 20 mV), the dissolution was triggered even in this region. This observation confirms the transient character of the cobalt dissolution, as it is mostly triggered by the significant change in the potential (ca. 50 mV), which causes surface restructuring due to either oxidation or reduction of the surface.

When we look at the pre-catalytic stabilization region, it seems that when the electrode is exposed to relatively constant conditions (or the slowly changing conditions at which the perturbations of the electrode/electrolyte interface are minimal, and the system has time to stabilize before the next bias change), the oxidation and reduction processes won't cause transient dissolution. Indeed, when comparing this trend with the one observed in Figure 1b, one can notice that higher dissolution rates during each OCP hold caused by the reduction of the Co_3O_4 surface correlate with constantly changing potential during these steps.

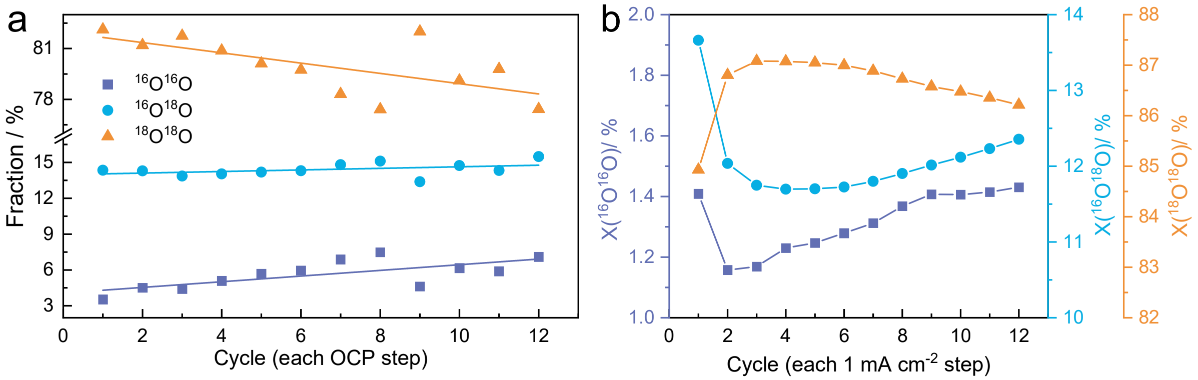

Figure S8. The fraction of $^{18}O^{18}O$ (m/z=36), $^{16}O^{18}O$ (m/z=34), and $^{16}O^{16}O$ (m/z=32) for each OCP hold (a) and each anodic hold (b).

Figure S8 presents the changes in the m/z=32 ($^{16}O^{16}O$), m/z=34 ($^{16}O^{18}O$), and m/z=36 ($^{18}O^{18}O$) fractions in the recorded signal separately during the OCP and the anodic holds. While the m/z=36 ($^{18}O^{18}O$) and m/z=34 ($^{16}O^{18}O$) signals during the OCP holds mostly come from the tailing of the intense signals during the previous anodic holds, the m/z=32 ($^{16}O^{16}O$) signal increases during each OCP hold (**Figure 3b** and Figure S8a). This correlates with the dissolution data and confirms our assumption about the liberation of oxygen from the lattice due to the reduction of the CoO_2 species and dissolution of Co (eq. C1, Chapter 3). The dissolution of the previously oxidized species should result in either a constant or increasing m/z=34 ($^{16}O^{18}O$) fraction if the lattice participates in the oxygen exchange. Indeed, the m/z=34 ($^{16}O^{18}O$) fraction slightly increases with the increased number of OCP cycles (Figure S8a). We can also say that this trend does not come from the tailing of the anodic signal, as m/z=36 ($^{18}O^{18}O$) and m/z=34 ($^{16}O^{18}O$) fractions demonstrate the opposite trends. The change in m/z=32 ($^{16}O^{16}O$), m/z=34 ($^{16}O^{18}O$), and m/z=36 ($^{18}O^{18}O$) fractions during the anodic holds is presented in Figure S8b, and correlates well with the overall trends presented in **Figure 3b**.

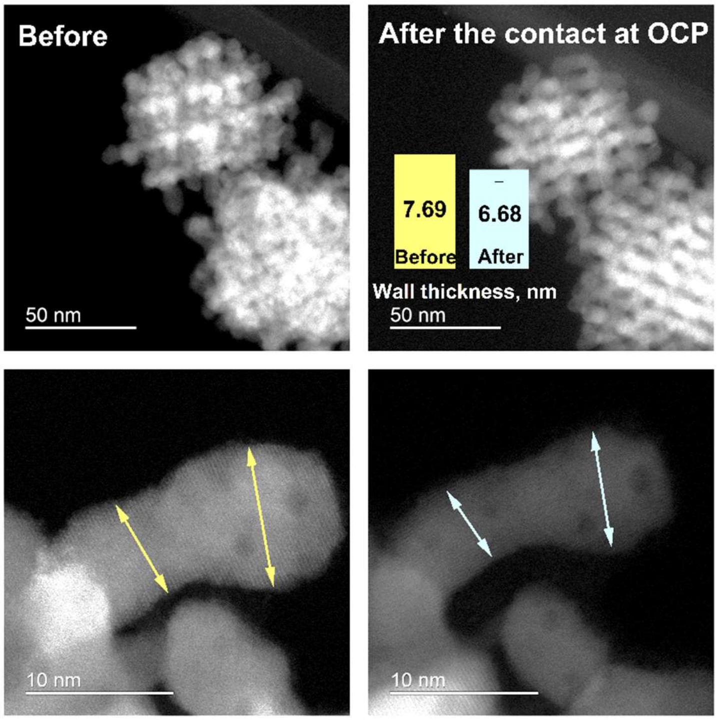

Figure S9. HR-IL-TEM images of Co_3O_4 before (left side) and after (right side) the contact of the sample with the electrolyte at OCP.

Chapter 3. Suggested reaction mechanisms and discussion.

In this chapter, we suggest possible mechanisms of cobalt dissolution due to multiple reactions occurring on the surface of Co_3O_4 in an acidic electrolyte when cyclic voltammetry is performed. We must underline here that all the presented equations are purely suggestions and are proposed here based on our results on cobalt dissolution obtained with online ICP-MS and production of water and oxygen data obtained with DEMS measurements. Further spectroscopic studies are needed to prove, refute, or improve the suggested mechanisms. Moreover, in some cases, we suggest a couple of alternative mechanisms. All these equations are summarized in Figure 5 (except for the alternative paths).

Possible reaction occurring at OCP (or at ca. 1 V_{RHE} due to the contact of the electrode with electrolyte), which causes the dissolution of Co:

$$Co^{II}Co_2^{III}O_4 + 4H^+ \rightarrow Co^{IV}O_2 + 2Co^{2+} + 2H_2O \ (OCP1)$$

$$2Co^{IV}O_2 + 4H^+ \rightarrow 2Co^{2+} + 2H_2O + O_2 \ (OCP2)$$

Our SFC-ICP-MS results demonstrate intense Co dissolution due to the initial contact with the electrolyte, and DEMS results suggest the formation of water and release of oxygen from the lattice due to this cobalt dissolution. According to Bloor et al.,[5] the equations OCP1 and OCP2 can describe the reactions taking place on the surface of cobalt oxides with no applied bias. It is worth noting, however, that the formation of $Co^{IV}O_2$ species at such low potentials is unlikely, according to the Pourbaix diagram (Figure S6). Therefore, the dissolution of cobalt with the formation of water and oxygen is either a two-step reaction, as described above, or there are some high oxidation state species like $Co^{IV}O_2$ present on the surface from the beginning. It could happen that there are trace amounts of such species formed during the synthesis or long exposure of the sample to air, as the DEMS technique is very sensitive. While we suggest these equations to describe the dissolution processes occurring during this step, we do not exclude the possibility that these processes are more complex and that there can be other ways to describe them.

A. Potential changes in the anodic direction:

Transient dissolution due to the oxidation reaction (A1 peak in Figure 2d):

$$Co^{II}Co_2^{III}O_4 + 2H^+ \rightarrow\ Co^{2+} +\ H_2O +\ Co_2^{III}O_3\ (A1)$$

$$Co^{II}Co_2^{III}O_4 + 2H^+ \rightarrow\ Co^{3+} +\ H_2O +\ Co_2^{III}O_3\ + e^-\ (A1.1)$$

As shown in Figure 2d, a small anodic peak (marked as A1) appears at the potential of ca. 1.3-1.55 V_{RHE}. Moreover, Figure 2a-b demonstrates that cobalt dissolution accompanies this oxidation (presumably of Co^{II} to Co^{III} species), highlighting its transient character. Equations A1 and A1.1 describe this reaction, where A1 is a chemical dissolution process, which could be coupled with the electrochemical process (A1.1). It can be written as one equation as follows:

$$2Co^{II}Co_2^{III}O_4 + 4H^+\ \rightarrow\ Co^{2+} + Co^{3+} +\ 2H_2O +\ 2Co_2^{III}O_3 +\ e^-(A1, alternative)$$

The dissolution of cobalt through the formation of Co^{3+} ions is questionable. However, this is the only possible way to balance the equation with our current knowledge.

Formation of stable $Co^{IV}O_2$ species in the pre-catalytic region due to further oxidation (A2 peak in Figure 2d):

$$Co_2^{III}O_3 +\ H_2O \rightarrow 2Co^{IV}O_2 + 2e^- + 2H^+\ (A2)$$

The formation of $Co^{IV}O_2$ species cannot be confirmed in this study, but to describe the mechanism, we assume their formation in a pre-OER region (ca. 1.55-1.65 V_{RHE} according to Figure 2). Moreover, according to the Pourbaix diagram (Figure S6), at higher local concentrations of dissolved Co ions, the formation of $Co^{IV}O_2$ is plausible. Here, no dissolution is observed.

B. Potential changes in the anodic direction – OER:

Lattice participation in OER:

$$2Co^{IV}O_2 + 2H_2O \rightarrow\ 2Co^{II}O + 4H^+ + 4e^- + 2O_2\ (B1)$$

Followed by Co dissolution:

$$Co^{II}O + H_2O \rightarrow\ Co^{2+} + 2H^+ + 4e^- + O_2\ (B1.1)$$

Or re-oxidation of cobalt species:

$$Co^{II}O +\ H_2O \rightarrow\ Co^{IV}O_2 + 2H^+ + 2e^-\ (B1.2)$$

Assuming the formation of $Co^{IV}O_2$ species in a precatalytic region, we then also assume their participation in the OER. Moreover, oxygen evolution occurs through lattice participation, as shown by the DEMS measurements (Figure 3). Therefore, we suggest the OER takes place via a

multi-step process described by equations B1, B1.1, and B1.2, where the first steps occur due to the oxidation of oxygen and reduction of cobalt species.

We also suggest an alternative path, which is presented below. The advantage of this alternative path is that it can easily be adapted for the Co^{II}/Co^{III} redox couple if further studies show no formation of the $Co^{IV}O_2$ species before or during the OER.

Alternative path of OER:

$$Co^{IV} - O^{2-} \rightarrow\ Co^{IV} - O\,\cdot^{-} +\ e^{-}\ (B2.1)$$

$$2\ (Co^{IV} - O\,\cdot^{-}) \rightarrow\ 2(Co^{III} - \blacksquare) + O_2\ (B2.2)$$

Followed by Co dissolution:

$$Co^{III} - \blacksquare \rightarrow\ Co^{2+} + e^{-}\ (B2.3)$$

Or re-oxidation of cobalt species:

$$2Co^{III} - \blacksquare + H_2O \rightarrow\ 2Co^{IV} - O^{2-} + 2\ H^{+} + 2e^{-}\ (B2.4),$$

where $O\,\cdot^{-}$ is a radical, and ■ is oxygen vacancy.

C. Potential changes in the cathodic direction:

Dissolution of Co due to the reduction of $Co^{IV}O_2$ species with simultaneous liberation of O_2 (e.g., start-up/shutdown operation, Figure 1b, C2 peak in Figure 2d):

$$2Co^{IV}O_2 + 4H^{+} \rightarrow\ 2Co^{2+} + 2H_2O + O_2\ (C2)$$

$$2Co^{IV}O_2 + 2e^{-} + 2H^{+} \rightarrow Co_2^{III}O_3 +\ H_2O\ (C2.1)$$

The first reduction peak of cobalt occurs at the potentials of ca. 1.65-1.55 V_{RHE} (Figure 2d, C2) and is accompanied by a moderate cobalt dissolution (Figure 2a-b). We speculate that previously formed (and not consumed in the OER) $Co^{IV}O_2$ species are now being reduced to Co^{III}-based species. Moreover, we suggest that this reaction can also be described as a combination of chemical and electrochemical processes.

Transient dissolution due to the reduction reaction (C1 peak in Figure 2d):

$$2Co_2^{III}O_3 + 4H^{+} + 2e^{-} \rightarrow\ Co^{II}Co_2^{III}O_4 + Co^{2+} + 2H_2O\ (C1)$$

A final step, the reduction of Co^{III}-based species to Co^{II}-based species on the surface and a return to the spinel structure, if possible, is described by equation C1. This process also initiates cobalt dissolution.

References

(1) Thommes, M.; Kaneko, K.; Neimark, A. V.; Olivier, J. P.; Rodriguez-Reinoso, F.; Rouquerol, J.; Sing, K. S. W. Physisorption of gases, with special reference to the evaluation of surface area and pore size distribution (IUPAC Technical Report). *Pure Appl Chem* **2015**, *87* (9-10), 1051-1069. DOI: 10.1515/pac-2014-1117.

(2) Schlumberger, C.; Thommes, M. Characterization of Hierarchically Ordered Porous Materials by Physisorption and Mercury Porosimetry-A Tutorial Review. *Adv Mater Interfaces* **2021**, *8* (4), 2002181. DOI: ARTN 200218110.1002/admi.202002181.

(3) Biesinger, M. C.; Payne, B. P.; Grosvenor, A. P.; Lau, L. W. M.; Gerson, A. R.; Smart, R. S. Resolving surface chemical states in XPS analysis of first row transition metals, oxides and hydroxides: Cr, Mn, Fe, Co and Ni. *Appl Surf Sci* **2011**, *257* (7), 2717-2730. DOI: 10.1016/j.apsusc.2010.10.051.

(4) Budiyanto, E.; Ochoa-Hernández, C.; Tüysüz, H. Impact of Highly Concentrated Alkaline Treatment on Mesostructured Cobalt Oxide for the Oxygen Evolution Reaction. *Adv Sustain Syst* **2023**, *7* (5). DOI: 10.1002/adsu.202200499.

(5) Bloor, L. G.; Molina, P. I.; Symes, M. D.; Cronin, L. Low pH Electrolytic Water Splitting Using Earth-Abundant Metastable Catalysts That Self-Assemble in Situ. *Journal of the American Chemical Society* **2014**, *136* (8), 3304-3311. DOI: 10.1021/ja5003197.
